# Temporal integration as “common currency” of brain and self**‐**scale‐free activity in resting‐state EEG correlates with temporal delay effects on self‐relatedness

**DOI:** 10.1002/hbm.25129

**Published:** 2020-07-22

**Authors:** Ivar R. Kolvoort, Soren Wainio‐Theberge, Annemarie Wolff, Georg Northoff

**Affiliations:** ^1^ Mind, Brain Imaging and Neuroethics Unit, Institute of Mental Health Research University of Ottawa Ottawa Ontario Canada; ^2^ Department of Psychology, Programme Group Psychological Methods University of Amsterdam Amsterdam The Netherlands

**Keywords:** common currency, EEG, scale‐free activity, self, self‐prioritization effect, self‐relatedness, temporal integration

## Abstract

The self is a multifaceted phenomenon that integrates information and experience across multiple time scales. How temporal integration on the psychological level of the self is related to temporal integration on the neuronal level remains unclear. To investigate temporal integration on the psychological level, we modified a well‐established self‐matching paradigm by inserting temporal delays. On the neuronal level, we indexed temporal integration in resting‐state EEG by two related measures of scale‐free dynamics, the power law exponent and autocorrelation window. We hypothesized that the previously established self‐prioritization effect, measured as decreased response times or increased accuracy for self‐related stimuli, would change with the insertion of different temporal delays between the paired stimuli, and that these changes would be related to temporal integration on the neuronal level. We found a significant self‐prioritization effect on accuracy in all conditions with delays, indicating stronger temporal integration of self‐related stimuli. Further, we observed a relationship between temporal integration on psychological and neuronal levels: higher degrees of neuronal integration, that is, higher power‐law exponent and longer autocorrelation window, during resting‐state EEG were related to a stronger increase in the self‐prioritization effect across longer temporal delays. We conclude that temporal integration on the neuronal level serves as a template for temporal integration of the self on the psychological level. Temporal integration can thus be conceived as the “common currency” of neuronal and psychological levels of self.

## Graphical abstract

The self‐integrates information and experience across multiple time scales. We here provide evidence for a relationship between temporal integration on both neuronal and psychological levels. Specifically, neuronal indices of temporal integration, power‐law exponent, and autocorrelation window, relate to psychological measures of temporal integration with respect to self. We therefore propose that temporal integration on the neuronal level serves as template or blueprint for temporal integration on the psychological level—temporal integration may serve as “common currency” of brain and self.

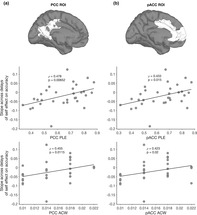

## 1 INTRODUCTION

### 1.1 Temporal integration and self‐psychological level

The “self” is a complex and multifaceted concept which includes a strong temporal dimension (Ersner‐Hershfield, Garton, Ballard, Samanez‐Larkin, & Knutson, 2009; Ersner‐Hershfield, Wimmer, & Knutson, 2008; Northoff, 2017; Wolff et al., 2019). One core temporal feature of the self is its ability to operate across, and thus integrate, different time scales, entailing temporal continuity (Ersner‐Hershfield et al., 2009; Ersner‐Hershfield et al., 2008; Northoff, 2017). While both the physiological and psychological aspects of our body and the environment change continuously, our self is characterized by stability and endures nonetheless—the self thus provides temporal continuity over both short and long timescales (e.g., Ersner‐Hershfield et al., 2009; Hershfield, 2011; Northoff, 2016, 2017; Schacter et al., 2012). The central importance of the temporal continuity of our self is further underlined by the fact that its disruption can lead to major alterations in our mental life, as manifested in psychiatric disorders like depression, mania, and psychosis or schizophrenia (Giersch & Mishara, 2017; Martin et al., 2014; Martin, Franck, Cermolacce, Coull, & Giersch, 2018; Martin, Giersch, Huron, & van Wassenhove, 2013; Northoff, 2007, 2014, 2016; Northoff et al., 2017). Together, the self can be characterized by temporal continuity across different time scales—the self is not bound to a specific time scale but is scale‐free (Huang, Obara, Davis IV, Pokorny, & Northoff, 2016; Wolff et al., 2019).

The self has been shown to modulate behavioral responses related to reward (de Greck et al., 2008, 2010; Yankouskaya, Bührle, Lugt, Stolte, & Sui, 2020), attention (Sui, He, & Humphreys, 2012; Sui, Rotshtein, & Humphreys, 2013; 2014; Humphreys & Sui, 2016), perception (Sui et al., 2012, 2013), emotion (Northoff et al., 2009; Yankouskaya et al., 2020), and decision making (e.g., Nakao et al., 2019; Nakao, Bai, Nashiwa, & Northoff, 2013; Nakao, Ohira, & Northoff, 2012). This has been described as the “integrative function of self” featured by “self‐expansion” of the self to different psychological functions (Northoff, 2016; Sui & Humphreys, 2015). Importantly, such integration and expansion is possible only by integrating the relatively long time scales of self, as manifest in its temporal continuity, and the much shorter time scales of various psychological functions and external stimuli (Northoff, 2017). What is described on the psychological level as the “integrative function of self” may thus, at least in part, be based on temporal integration of these different time scales (Himberger, Chien, & Honey, 2018; Northoff, 2017). However, such temporal integration of different time scales on the psychological level of self remains yet to be experimentally probed.

### 1.2 Temporal integration and the brain

The brain's spontaneous activity exhibits oscillatory patterns, ranging from ultrafast frequencies (40–180 Hz) to infraslow ones (0.01–0.1 Hz; Buzsáki, 2006; Buzsáki & Draguhn, 2004). The power in these frequency ranges increases with lower frequencies. Hence, the power is strongest in the slowest frequencies and weakest in the faster frequencies. It has been found that this pattern follows a power law distribution (see below for details; Eke et al., 2000; Eke, Herman, Kocsis, & Kozak, 2002; He, 2011, 2014; He, Zempel, Snyder, & Raichle, 2010; Hiltunen et al., 2014; Huang et al., 2016; Linkenkaer‐Hansen, Nikouline, Palva, & Ilmoniemi, 2001). The relation between different frequencies of neuronal activity can be described as being scale‐free or scale‐invariant due to the fact that the relationship between the power of the frequencies is the same regardless of what range of frequencies one looks at (Chialvo, 2010; Eke et al., 2000, 2002; He, 2011, 2014; He et al., 2010).

Scale‐invariance is closely related to temporal integration. Scale‐invariance denotes that statistical properties of a signal (in this case, the relationship between power in different frequencies) are constant no matter the size of the time scale examined, as such it signifies a “continuity” of the neuronal activity analogous to the self's continuity over different timescales discussed above. Furthermore, scale‐free activity in the brain is strongly associated with connections between high and low frequencies through mechanisms such as cross‐frequency coupling (He, 2014; He et al., 2010). The importance of scale invariance as a marker of neuronal activity is evidenced by the fact that alterations in the scale‐free neuronal activity have been related to disorders such as Alzheimer's disease (Maxim et al., 2005), schizophrenia (Sokunbi et al., 2014), and autism spectrum disorder (Damiani, Scalabrini, Gomez‐Pilar, Brondino, & Northoff, 2019; Dona, Hall, & Noseworthy, 2017).

This scale‐invariance or self‐similarity can be measured in the frequency domain and expressed by

$$P\propto 1/f^{\beta }$$

*P* stands for power, which is proportional to the inverse of the frequency (*f*) raised to the power *β* (Eke et al., 2000, 2002; He, 2011, 2014). Throughout this paper we refer to *β* as the power‐law exponent (PLE) in order to avoid confusion with the *β* symbol we use for regression coefficients later in the paper. This PLE parameter indicates the degree of self‐similarity: a value of 0 for this parameter would indicate no structure being present, as in a white‐noise signal (He, 2014). We use the terms “scale‐free,” “scale‐invariant,” “fractal scaling,” and “self‐similarity” to all refer to this same inverse‐power‐law relationship between power and frequency.

A closely related measure is the autocorrelation window (ACW). ACW measures temporal integration in the time domain by way of correlations across different time points (Gollo, Roberts, & Cocchi, 2017; Gollo, Zalesky, Hutchison, van den Heuvel, & Breakspear, 2015; Himberger et al., 2018; Honey et al., 2012; Murray et al., 2014). To compute the ACW, first an autocorrelation function is constructed by correlating a time series with copies of itself, shifted in time with various lags. This results in a correlation as a function of time lag. The autocorrelation is one at lag zero, and decays at larger lags as the shifted copies become less correlated with the original. The “width” of the autocorrelation function (i.e., the lag value at which the autocorrelation function reaches 0.5) measures how consistent the time series is from time point to time point. ACW and PLE asses the same property and are equivalent in the case of synthetic scale‐free signals (Eke et al., 2000, 2002). However, EEG signals contain numerous nonscale‐free processes as well as various neuronal (and non‐neuronal) noise sources, and these measures may be differentially affected by these processes. Hence, we include both measures as indices of temporal integration in this study.

### 1.3 From brain to self‐temporal integration as their “common currency”

Recent studies in both fMRI (Huang et al., 2016) and EEG (Wolff et al., 2019) show that interindividual variation in resting‐state PLE explains a high degree of interindividual variation in self‐consciousness. Higher PLE values in resting‐state neuronal activity are related to higher degrees of self‐consciousness. The same EEG study observed that the ACW also correlated with the degree of private self‐consciousness of an individual (Wolff et al., 2019).

These data suggest that PLE and ACW, as indexes of temporal integration on the neuronal level (Gollo et al., 2015, 2017; Himberger et al., 2018; Murray et al., 2014), are related to self‐consciousness. In addition to measures of self‐consciousness, fMRI research has shown that individual differences in resting‐state PLE in cortical midline regions are related to individual differences in task evoked activity in response to self‐related animate stimuli, but not to inanimate stimuli (Scalabrini et al., 2017, 2019). This implies a form of self‐specificity of fractal structure in brain activity. The aforementioned relationship of PLE with self‐consciousness may also be particularly strong specifically in cortical midline regions, such as the perigenual anterior cingulate cortex (pACC) and posterior cingulate cortex (PCC), as these areas are known to be involved in self‐related processing (Murray, Debbane, Fox, Bzdok, & Eickhoff, 2015; Northoff et al., 2006; Northoff & Bermpohl, 2004; Van der Meer, Costafreda, Aleman, & David, 2010).

More generally, Scalabrini, Mucci, and Northoff (2018) have suggested that studying how resting‐state activity related to the self‐interacts with external stimuli, that is, rest/self‐stimulus interaction (Northoff, Qin, & Nakao, 2010), can clarify the functional role of spontaneous brain activity for behavioral outcomes. However, specifically whether resting‐state temporal integration of different time scales on the neuronal level mediates temporal integration on the psychological level of self (with temporal integration providing their “common currency”; Northoff, Wainio‐Theberge, & Evers, 2020), remains unclear.

### 1.4 Aims and hypotheses

The main and overarching aim in the present study is to investigate and connect temporal integration of self on both psychological and neuronal levels. We hypothesized that temporal integration on the psychological level of self, as tested by investigating the effects of temporal delays on self‐specificity judgments in the self‐matching task, is related to temporal integration on the neuronal level of the brain's resting‐state, as measured by PLE and ACW. For that purpose, we recruited a group of healthy subjects to undergo an EEG resting‐state measurement and participate in a behavioral experiment targeting temporal integration.

The first specific aim consisted of investigating temporal integration on the psychological level of self. We here rely on the well‐established perceptual matching task (Sui et al., 2012), which focuses specifically on the integrative function of self: subjects are required to connect and thus integrate two different stimuli (labels and geometric shapes) in order to judge whether they match. This typically has shown more efficient processing for self‐related stimuli, measured as increased accuracy or decreased response times, which has been termed the self‐prioritization effect (SPE; Sui & Humphreys, 2015). Following our focus on specifically the temporal integration of self, we modified the perceptual matching paradigm by including a temporal component in the form of different delays between the presentation of shape and label. Accurate responses require one to integrate the two stimuli over time whereas inaccurate responses indicate a failure of this temporal integration. Based on the SPE we hypothesized a higher level of accuracy in the self conditions and that this advantage would be maintained over the different temporal delays. To assess the role of cognitive functioning in this process, we also measured working memory and assessed learning effects.

The second specific aim was to investigate temporal integration on the neuronal level and how that relates to temporal integration of self on the psychological level. For that purpose, we recorded resting‐state EEG and used the above‐mentioned measures, ACW and PLE, as indices of temporal integration on the neuronal level. In recent theoretical work it has been proposed that interindividual differences in self‐related resting‐state activity related are crucial for explaining individual differences in behavioral outcomes (Scalabrini et al., 2018). Based on this and previous empirical research (Huang et al., 2016; Wolff et al., 2019), we hypothesized that interindividual variation in resting‐state ACW and PLE in specifically midline regions like pACC and PCC is related to interindividual variation in changes of the SPE over the different temporal delays. Specifically, we hypothesized that higher degrees of temporal integration on the neuronal level, that is, higher PLE and longer ACW, are related to larger SPEs over the longer temporal delays. We hypothesized that this relationship is not related to cognitive function, that is, working memory and learning effects.

## 2 METHOD

### 2.1 Participants

Thirty‐one adolescents (mean age = 20.4 years, *SD* = 1.77; 17 females) participated in this study. Participants were recruited primarily from the University of Ottawa, and from the general population. The study took place at The Royal Ottawa's Institute for Mental Health Research and lasted approximately 2 hr per participant, for which they were financially compensated. The study was approved by The Royal Ottawa Research Ethics Board (REB#2017019) and all participants provided written informed consent prior to participation.

### 2.2 Apparatus, stimuli, and procedure

#### 2.2.1 EEG resting‐state measurement

First participants underwent an EEG resting‐state measurement. For this they were required to look at a gray screen with a white fixation cross for 7 min. Participants were instructed to keep their eyes open and to keep head movements to a minimum.

#### 2.2.2 Perceptual matching task

The perceptual matching task we used was a modified version of the one developed by Sui, He, and Humphreys (Sui et al., 2012). Instructions were presented on screen and afterward participants completed four blocks, each consisting of a learning and testing stage.

For each block, three geometrical shapes (circle, square, and triangle) were assigned randomly to three personal labels (“You,” “friend,” and “stranger”). These were presented as shape‐label pairs to the participants in text during the learning stage. Participants could take as much time as they needed to memorize these associations (average was 2 min). Afterward participants performed 100 trials in which a shape‐label pair was presented. The task for the participants was to judge whether the presented pair was correct according to the associations memorized in the learning phase.

**FIGURE 1:**
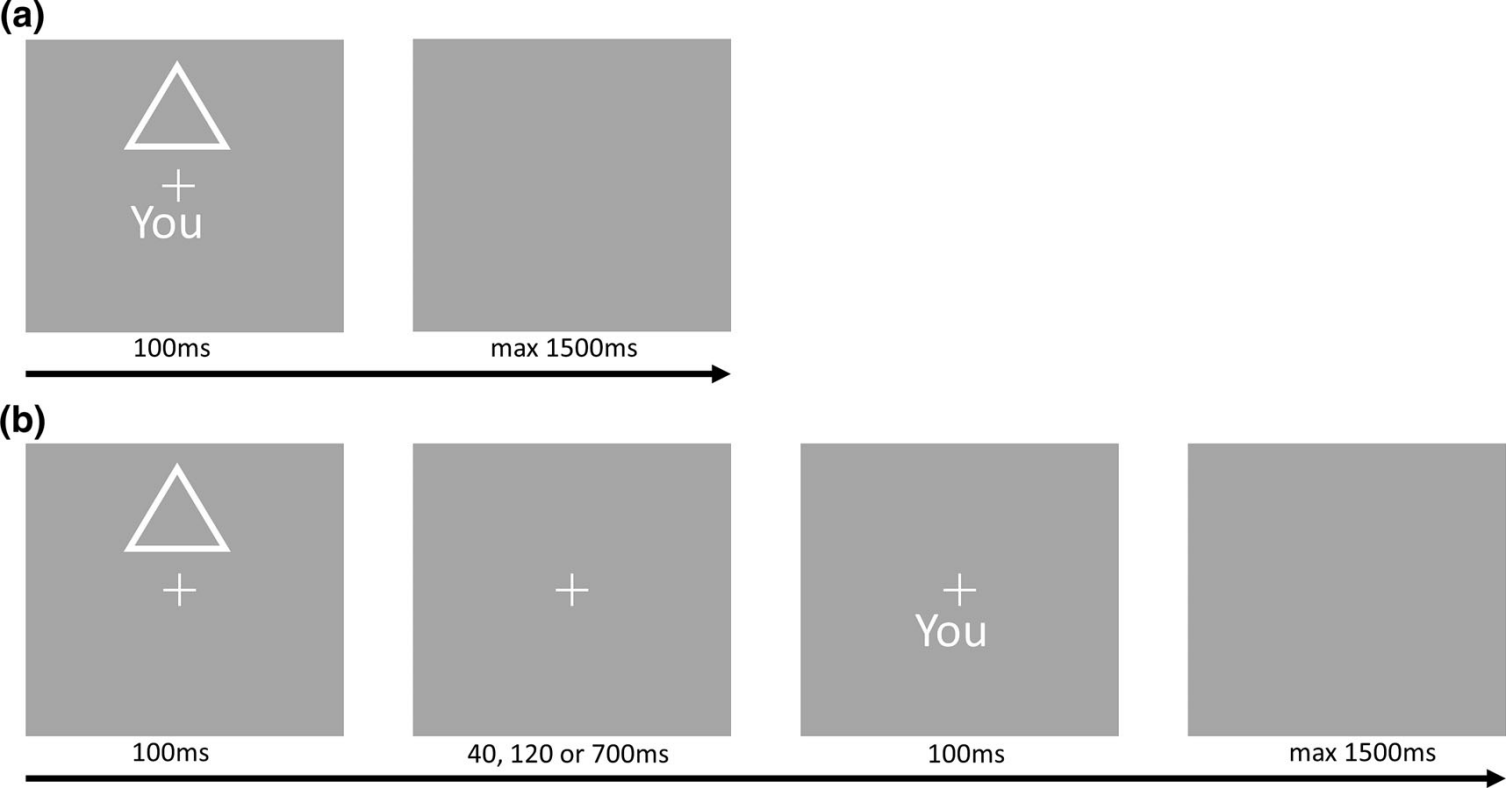
Example of experimental stimuli. (a) Example of stimuli in the no delay condition. The response screen lasts up to 1,500 ms and is terminated by a response. (b) Example of stimuli in the delay conditions. The second screen is visible during the delay

There were four conditions based on the delays between shape and label. First, shape and label were presented simultaneously (no delay) for 100 ms. Next, there were three different delays present after the presentation of the shape and before the presentation of the label. The delays were 40, 120, and 700 ms during which a central fixation cross was displayed. We chose these delays as they represent approximately one cycle length of the beta, alpha, and delta EEG frequency bands, which have been found to be central in analyzing human EEG data (Niedermeyer & da Silva, 1999). Moreover, these delays are similar to the ones used by Janczyk and colleagues (50–1,000 ms) in their investigation of the SPE (2018). These four conditions were randomized (Figure 1).

All trials consisted of the following sequence: a jittered intertrial interval (2–4 s, steps of 0.5 s) with a central fixation cross. Then the shape‐label pair was presented. In the no delay conditions both shape and label were presented together for 100 ms. In the delay conditions the shape was presented first for 100 ms and after a delay (40, 120, or 700 ms) the label was presented for 100 ms. After the label was presented, a blank screen was shown for a maximum of 1,500 ms during which participants could respond.

Participants were instructed to respond as quickly as possible. When they failed to do so within the 1,500 ms limit, the trial was registered as missing and the next trial started. This was done to ensure rapid, spontaneous responses. Participants responded “correct” or “incorrect” on each trial using the “n” and “m” keys on the keyboard; which key corresponded to each response was counterbalanced across participants.

All shape‐label stimuli were generated randomly. Hence, we had three within‐subject variables: Self (self‐associated shape or nonself, that is, friend or stranger, associated shape), Matching (matching shape‐label pair with learned associations or nonmatching) and Delay (no delay, 40, 120, or 700 ms). This design resulted in 2 × 2 × 4 = 16 conditions.

### 2.3 Data analyses

#### 2.3.1 Preprocessing of behavioral data

First we removed trials where the reaction time (RT) was shorter than 200 ms (Ratcliff, 1993). Then, as a standard way of dealing with outliers, we removed trials where the RT was lower or higher than 2 standard deviations from the mean RT of each participant within each condition. In total this removed 5% of our data, well within the standards set for outlier removal in RT analysis (Ratcliff, 1993). For the analysis of efficiency scores (see below) we used this dataset. For the analysis of RTs, using a linear mixed effects regression model (henceforth LMM), only the correct responses were included. For the separate analysis of accuracy using a logistic mixed effects model (henceforth LogMM) we did not remove outliers based on RT (as they are not outliers in accuracy) and recoded missed observations as incorrect.

#### 2.3.2 Bootstrapping

We used a bootstrapping procedure in order to get a sense of the central tendency of the responses (Davison & Hinkley, 1997; Sui et al., 2012). For each condition 250 bootstrapped samples were created by resampling the data with replacement. For each of these samples the mean RT and accuracy were calculated and plotted in order to visualize both the mean and variance of the data for each condition.

#### 2.3.3 Efficiency scores

We calculated efficiency scores for each participant within each condition by dividing the mean RT by the proportion of correct responses (Sui et al., 2013; Townsend & Ashby, 1983). This provided us with a measure combining both aspects of participants' responses (accuracy and RT) that indicates how efficient participants are in providing accurate responses. For this measure lower values indicate more efficient responses. Paired samples t‐tests were used on order to examine the within‐subject effects *on efficiency scores*.

#### 2.3.4 Mixed effects model regressions (accuracy and response times)

We analyzed accuracy and RTs separately using a mixed effects regression approach. The mixed effects approach used here has several advantages compared with, for example, ANOVA, including the ability to flexibly calculate contrasts and to deal with unbalanced repeated measures data (see Gueorguieva & Krystal, 2004; Quené & Van den Bergh, 2008). We added subject into the model as a random effect, a so‐called “random intercept model,” which allows the model to estimate a separate intercept for each participant and estimates the effects of other variables relative to each individual's mean response. In addition, we added all other experimental variables (Self, Matching, and Delay) into the regression as fixed effects. We estimated full factorial models, meaning that we estimated the main effects of the experimental variables and all their interactions in the regression models. This analysis was done using the lme4 package in R (Bates, Mächler, Bolker, & Walker, 2014, RRID:SCR_015654).

Lastly, with this approach we can apply a similar analysis to both RT and accuracy. For RT we will simply estimate a linear model (i.e., a linear mixed model, henceforth LMM). Since accuracy is a binary dependent variable, having a value of 0 for incorrect responses and a value of 1 for correct responses, we use a logistic model for the accuracy data (Hosmer Jr, Lemeshow, & Sturdivant, 2013). Such a model uses a logistic function to transform the predictions of the model into a continuous distribution. In our case this distribution then represents the probability a participant provides a correct response (accuracy = 1). Hence the model we used is a logistic mixed effects model (henceforth LogMM).

In all regression analyses we use the variables Matching and Self, where nonmatching and nonself conditions assign the variable as a value of 0, while the matching and self conditions are coded with a value of 1. Hence, the coefficients of these variables can be interpreted as the effect on the dependent variable of going from nonmatching to matching conditions (i.e., the matching effect), and as the effect of going from nonself to self conditions (i.e., the SPE) respectively. After first analyzing the behavioral responses in just the no delay conditions, we will apply the model to all conditions and include a variable Delay. The variable Delay was treated as a categorical variable and dummy coded for the regression analyses.

In contrast to Sui and colleagues (e.g., Sui et al., 2012; Yankouskaya et al., 2020), we do not perform our analysis of RT and accuracy separately for the matching and nonmatching conditions. Instead, we included a variable for Matching in our regressions so that we can explore its interaction terms with other variables. Ultimately, we used the estimated regression coefficients of the variables and (polynomial) contrasts comparing different levels of the variables to make inferences. Residual plots of all regression models were inspected for violations of homoscedasticity and normality.

#### 2.3.5 Controlling for cognitive effects: Working memory and learning

In order to control for working memory effects, participants did the operation span task after the perceptual matching task. Based on the operation span task, we calculated each individual's working memory score using the partial‐credit load scoring procedure (Conway et al., 2005). Based on this we performed a median split, creating a high and low WM group, and then compared their efficiency scores using t‐tests. To investigate the effect of working memory on accuracy and RT separately we added the working memory scores as a continuous covariate to our regression models. We added the main effect of working memory and its interactions with the self and delay variable.

To test whether the SPE is influenced by learning and practice, we used the trial number within each block as a covariate. As participants had to learn new associations each block, learning could only affect behavior over the duration of a single block. We analyzed efficiency scores, RTs, and accuracy separately. We calculated efficiency scores for each participant separately for the first and last half of each block and compared the different conditions using paired *t*‐tests. For accuracy and RT we included within‐block trial number as a covariate in the regression analyses.

### 2.4 EEG data acquisition and analysis

EEG data was recorded using Ag/AgCl electrodes through a 64‐channel Brain Vision Easycap (according to the International Ten‐Twenty System) referenced to the right mastoid. The data was sampled at 1,000 Hz with DC recording. The EEG data preprocessing was performed using the EEGLAB toolbox for MATLAB (version 14.1.1; Delorme & Makeig, 2004, RRID:SCR_007292). The data was downsampled to 250 Hz and filtered with a low‐pass filter at 50 Hz and a high‐pass filter at 0.5 Hz. Next, the data was cleaned using artifact subspace reconstruction (EEGLAB plugin clean_rawdata) to remove noisy channels (Chang, Hsu, Pion‐Tonachini, & Jung, 2018). Further artifacts were removed using independent component analysis (ICA), performed using the EEGLAB software creating 62 independent components. Next, we used the MARA implementation to automatically reject noisy components (Winkler, Haufe, & Tangermann, 2011). All removed channels were then spherically interpolated and all channels were re‐referenced to the average. Ten participants initially used a different electrode cap in order to record functional Near Infrared Spectroscopy (fNIRS) data; for these participants, any missing Easycap electrodes were interpolated following preprocessing, and additional electrodes from the fNIRS cap were removed.

The PLE was calculated using the enhanced periodogram method described in Eke et al. (2000), termed ^low^PSD_w,e_. Parabolic windowing and endmatching (described in detail in Eke et al., 2000) were first applied to the EEG signal. The power spectral density (PSD) was then estimated with Welch's method (Welch, 1967), and MATLAB's *polyfit* function was used to fit a line to the power spectrum in the range of our bandpass (0.5–50 Hz). The negative of the slope of this line was then extracted as the PLE, representing the relationship between power and frequency. One PLE value was extracted for each channel and specific regions of interest (ROIs; see below).

In order to make our PLE calculation more robust, we used a recently developed method, Irregular Resampling for Auto‐Spectral Analysis (IRASA; Wen & Liu, 2016), to separate fractal and oscillatory components in the power spectrum. This allows us to associate SPE with specifically the fractal component of the power spectrum, that is, the fractal spectrum, as distinguished from the oscillatory component and the nonseparated power spectrum, the latter of which we will refer to hereafter as the mixed spectrum. Oscillations are a widely studied and salient feature of EEG data (Buzsáki, 2006). However, when attempting to assess scale‐free activity, these oscillations are not of interest and instead can bias the estimation. The IRASA method separates these components by resampling the signal by a number of noninteger factors. Scale‐free activity is consistent across these samples, while oscillations with specific frequencies are shifted. The median of the resampled power spectra is taken as the fractal component, while the residual power is taken as reflecting scale‐dependent oscillatory processes. Following a previous study (Muthukumaraswamy & Liley, 2017), we computed the IRASA power spectrum in 10‐s windows with no overlap, using resampling factors ranging from 1.1 to 2.9 in steps of 0.05 (excluding 2). Due to the presence of filtering artifacts in the fractal PSD (see Figures S4 and S5), the IRASA based PLE, henceforth fractal‐spectrum PLE, was estimated across a smaller range of frequencies (2–25 Hz) than the mixed‐spectrum PLE.

To ensure robustness of our findings in relation to temporal integration, we employed an additional index of temporal integration; the ACW. As pointed out in the introduction, ACW (Himberger et al., 2018; Honey et al., 2012; Murray et al., 2014) and PLE (Chialvo, 2010; Eke et al., 2000, 2002; He, 2011, 2014; He et al., 2010; Linkenkaer‐Hansen et al., 2001) are analogous measures of temporal integration on the neuronal level; they assess the same property (the fractal scaling of power vs. frequency) in the frequency (PLE) and time (ACW) domains. These two ways of viewing temporal integration, through the lenses of frequency and time, are mathematically equivalent—the Wiener–Khinchin theorem relates the autocorrelation function, used to calculate the ACW, to the power‐spectrum, used to calculate the PLE.

The ACW was calculated according to the methods of Honey et al. (2012). The autocorrelation function was calculated on 20 s segments of the EEG signal, with 50% overlap. For each segment, the full width at half maximum of the autocorrelation function was calculated, and these values were averaged over all 20‐s segments to produce the ACW value for each electrode and ROI.

### 2.5 Regions of interest

As previous studies highlight the central role of resting‐state activity in cortical midline structures like pACC and PCC for the self (Northoff & Bermpohl, 2004; Northoff et al., 2006; van den Meer et al., 2010; Qin & Northoff, 2011; Murray et al., 2015; Huang et al., 2016; Wolff et al., 2019), we specifically analyzed ACW and PLE in these regions.

In order to create timeseries for those specific ROIs we used exact Low Resolution Electromagnetic Tomography (eLORETA, RRID:SCR_013830). eLORETA was used to compute the 3D intracerebral distribution of sources of scalp‐recorded electrical potentials (Pascual‐Marqui, 2007; Pascual‐Marqui, Michel, & Lehmann, 1994). ROIs were defined using the software's default Brodmann atlas: pACC (Brodmann areas 10, 24, 32) and PCC (Brodmann areas 23, 29, 30, 31). We also computed time courses in three control regions: primary visual cortex (Brodmann area 17), primary somatosensory cortex (Brodmann areas 1–3), and primary motor cortex (Brodmann area 4). Since the resultant time courses for each ROI reflect non‐negative current estimates, we log‐transformed them and subtracted the mean to make the time series more stationary and Gaussian to improve estimation (Cramér, 1992). We then computed mixed‐spectrum PLE and ACW according to the methods described above. Fractal‐spectrum PLE was not computed, as no prominent oscillations were observed in the eLORETA time series.

### 2.6 Relationship between behavioral delay effects and scale‐free activity in resting‐state EEG

Scale‐free activity in the brain, as measured by PLE and ACW, can be mechanistically related to behavioral effects with respect to time (Kello et al., 2010; Linkenkaer‐Hansen et al., 2001; Northoff, 2017; Palva et al., 2013). Hence, we looked at the change in SPE due to the delays as this captures how the effects of self and time interact. To capture the individualized patterns of change in the SPE, that is, how the SPE was modulated by introducing delays, we plotted each participant's mean SPE value as a function of the delay and then for each participant fitted a line to their SPE values by minimizing the squared error. The slope of this line, referred to henceforth as “SPE slope,” indicates the change of the SPE due to the delays: a positive slope in this line indicates that a participant's SPE increases with longer delays, while a negative slope indicates that a participant's SPE decreases when the delays are longer. This slope was used for the correlation analyses with the neuronal data. We computed Spearman correlations at each electrode and used a cluster‐based permutation test (Maris & Oostenveld, 2007) to correct for multiple comparisons. With the cluster‐based permutation tests we report the correlation coefficient computed from the average signal of all channels. Additionally, we employed partial correlations to control for cognitive effects due to working memory and learning differences.

To further ensure that our methodology is appropriate and our findings robust, we performed several control analyses. We calculated the above correlations with PLE and ACW computed over different frequency ranges, correlated the three measures of fractal structure (mixed‐spectrum PLE, fractal‐spectrum PLE, and ACW), and controlled the SPE slope correlations for parameters describing each subject's individual alpha peak. These control analyses are reported in Figures S1‐S3.

## 3 RESULTS

### 3.1 Psychological level of self I: No delay condition

#### 3.1.1 Efficiency scores

First, we analyzed only the trials in which there was no delay between the presentation of shape and label. Age and sex were found not to affect these results, nor do they affect any of the results mentioned later in the text, and as such they are not discussed further. Regarding only the no delay condition, there are two within‐subject variables: Self (self/friend/stranger associated shape) and Matching (matching/nonmatching shape‐label pair).

**FIGURE 2:**
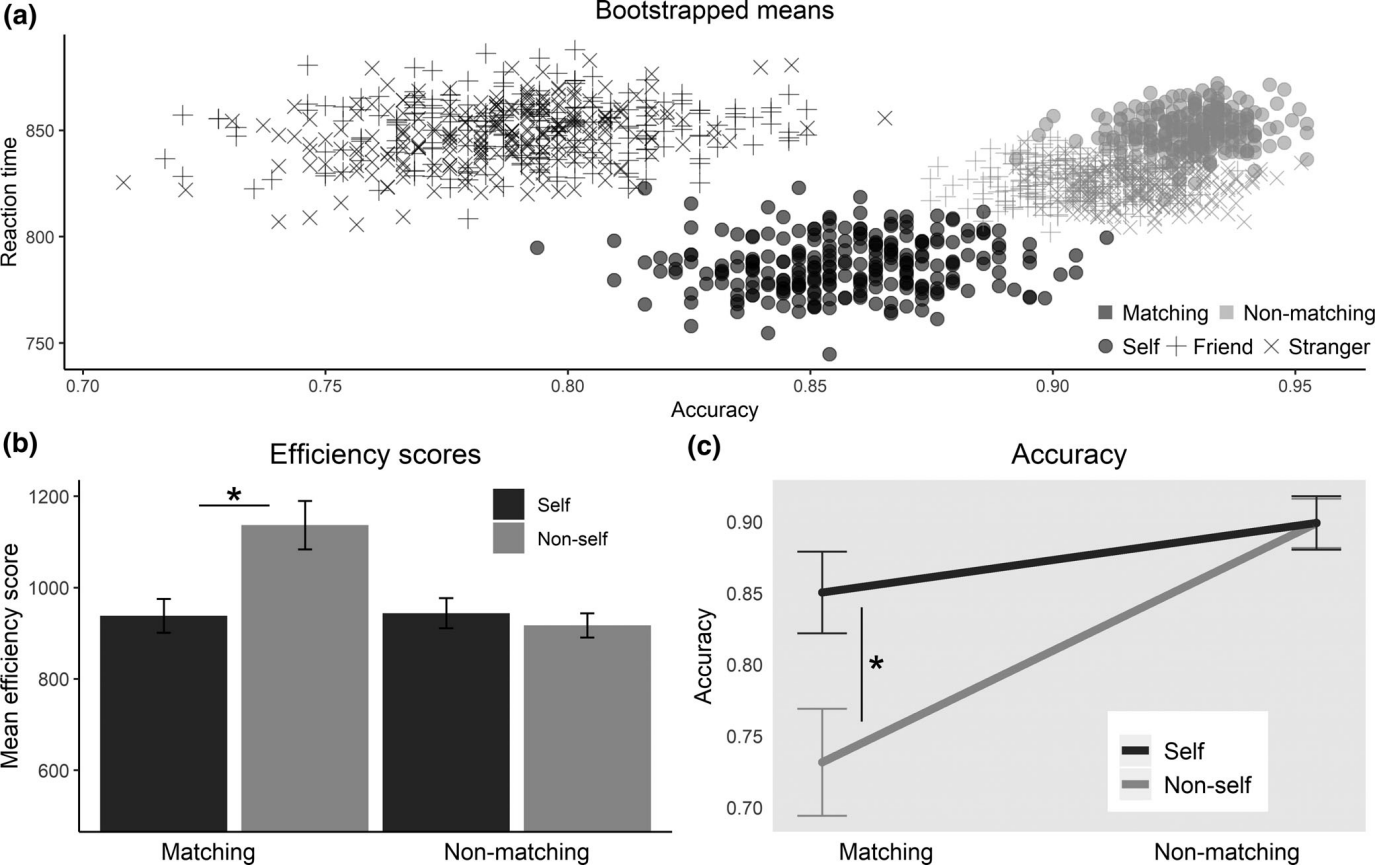
Effect of self and matching without delays. Based only on trials without a delay. Asterisks indicate *p* < .05. (a) Bootstrapped mean values of accuracy and reaction time, using 250 samples per condition sampled with replacement. (b) Mean efficiency scores (reaction time divided by accuracy), bars indicate standard errors. (c) LogMM predictions of the probability that a response is correct. Bars represent standard errors of predictions

Each point in Figure 2a represents the mean RT and accuracy of a bootstrapped sample. The figure shows a clear SPE on both accuracy and RT in the matching conditions, but not in the nonmatching conditions. Moreover, it shows that the friend and stranger means and variances are similar in both the matching and nonmatching conditions. Based on this and the fact that we are interested primarily in the self, we grouped friend and stranger together as “nonself” in subsequent analyses.

The mean efficiency scores (mean RT divided by the proportion of correct responses) for each condition are displayed in Figure 2b. Paired sample t‐tests show that the SPE in the matching conditions is highly significant *t*(30) = 3.55, *p* = 0.0013 (mean difference = 198.6), but not in the nonmatching conditions (*t*(30) = −1.173, *p* = 0.25, mean difference = −26.6).

#### 3.1.2 Accuracy

We then analyzed accuracy separately by fitting a logistic mixed effects regression model, with self and matching and their interaction as fixed factors and random intercepts by participant. Both the main effect of matching (*β* = −1.182, *SE* = 0.127, *p* < .001) and the interaction effect of self and matching (*β* = .733, *SE* = 0.229, *p* = .001) are highly significant. In contrast, the SPE was not significant as a main effect over both matching and nonmatching conditions (*β* = .00267, *SE* = 0.146, *p* = .985). The interaction effect of self and matching on accuracy is displayed in Figure 2c, where the model predictions of mean accuracy are presented together with their standard error.

Using post hoc analysis to confirm that the SPE is present in the matching conditions, but not in the nonmatching conditions, we estimated the marginal effects of self separately in the matching and non‐matching conditions with Bonferroni correction. The coefficient was highly significant in the matching condition (*β* = .116, *SE* = 0.0272, *p* < .001), but not in the nonmatching conditions (*β* = .0003, *SE* = 0.015, *p* = 1.00). This indicates that in the matching conditions participants responded more accurately when the presented shape was associated to themselves as compared to a friend or a stranger, confirming the existence of an SPE on accuracy.

#### 3.1.3 Reaction times

Next, we analyzed RTs separately by fitting a linear mixed effects regression model, again with self and matching and their interaction as fixed factors and random intercepts by participant. The SPE was marginally significant as main effect over both matching and nonmatching conditions (*β* = 17.37, *SE* = 9.46, *p* = .066), while, similar as with accuracy, the main effect of matching was significant (*β* = 31.35, *SE* = 10.37, *p* = .003), as was the interaction effect (*β* = −89.50, *SE* = 17.22, *p* < .001).

We did a similar post hoc analysis for RTs as done on accuracy. We find that, again, using Bonferroni correction, the SPE is significant in the matching conditions (*β* = −72.13, *SE* = 14.36, *p* < .001), but not in the nonmatching condition (*β* = 17.37, *SE* = 9.46, *p* = .133). This confirms the finding by Sui and colleagues that there is a SPE on RTs, where people respond more quickly to self‐related stimuli in the matching conditions (Sui et al., 2012).

### 3.2 Psychological level of self II: Effect of delays

#### 3.2.1 Efficiency scores

Next, we analyzed the influence of the delays on participant's responses. First, we calculated efficiency scores for each participant separately for each delay. We see that the mean efficiency scores decrease with increased delays: no delay (*M* = 950.9, *SD* = 123.1), 40 ms (*M* = 846.0, *SD* = 123.3), 120 ms (*M* = 815.3, *SD* = 137.9), 700 ms (*M* = 696.2, *SD* = 153.1). To test whether the efficiency scores in the conditions with a delay were significantly different we did multiple paired *t*‐tests with Bonferroni correction comparing each delay with the previous one. The difference between no delay and 40 ms is significant (*t* = 8.21, *p* < .001), as are the differences between 40 and 120 ms (*t* = 3.16, *p* = .006) and between 120 and 700 ms (*t* = 10.0, *p* < .001).

#### 3.2.2 Accuracy

We then analyzed accuracy separately by fitting a logistic mixed effects regression model (LogMM) as specified before. With regard to the main effect of the delays, we find that the effect of the 40 ms delay is not significantly different from the no delay condition (*β* = .248, *SE* = 0.133, *p* = .062). In contrast the 120 ms and 700 ms delay conditions are significantly different from the no‐delay condition (120 ms: *β* = .296, *SE* = 0.132, *p* = .025.; 700 ms: *β* = .700, *SE* = 0.141, *p* < .001). This indicates participants become more accurate with increasing delays. We also observed significant effects of self in delay conditions (*β* = .744, *SE* = 0.188, *p* < .001), indicating that participants were more accurate in response to self‐related stimuli.

#### 3.2.3 Reaction time

RTs were analyzed separately by fitting a linear mixed effects regression model (LMM). The model specification was with same factors (2 × 4 full factorial) as the above logistic regression model for accuracy. The overall SPE is not significant (*β* = −17.94, *SE* = 19.9, *p* = .088). With regard to the main effect of the delays, we find that the effect of the 40 ms delay is not significantly different from the no delay condition (*β* = −25.10, *SE* = 16.37, *p* = .13). In contrast, the 120 and 700 ms delay conditions are significantly different from the no delay condition (120 ms: *β* = −50.4, *SE* = 16.3, *p* = .002; 700 ms: *β* = −164.5, *SE* = 16.5, *p* < .001), indicating that participants respond quicker when longer delays are included between shape and label.

Together these results show that increasing the delay between the presentations of shape and label increases accuracy and shortens RTs. Importantly, the SPE of the no‐delay condition was preserved in and thus carried over to the delay conditions. We then focused our investigation on the interaction of self and temporal delay, the subject of our hypothesis.

### 3.3 Psychological level of self III: Interaction of self and delays

**FIGURE 3:**
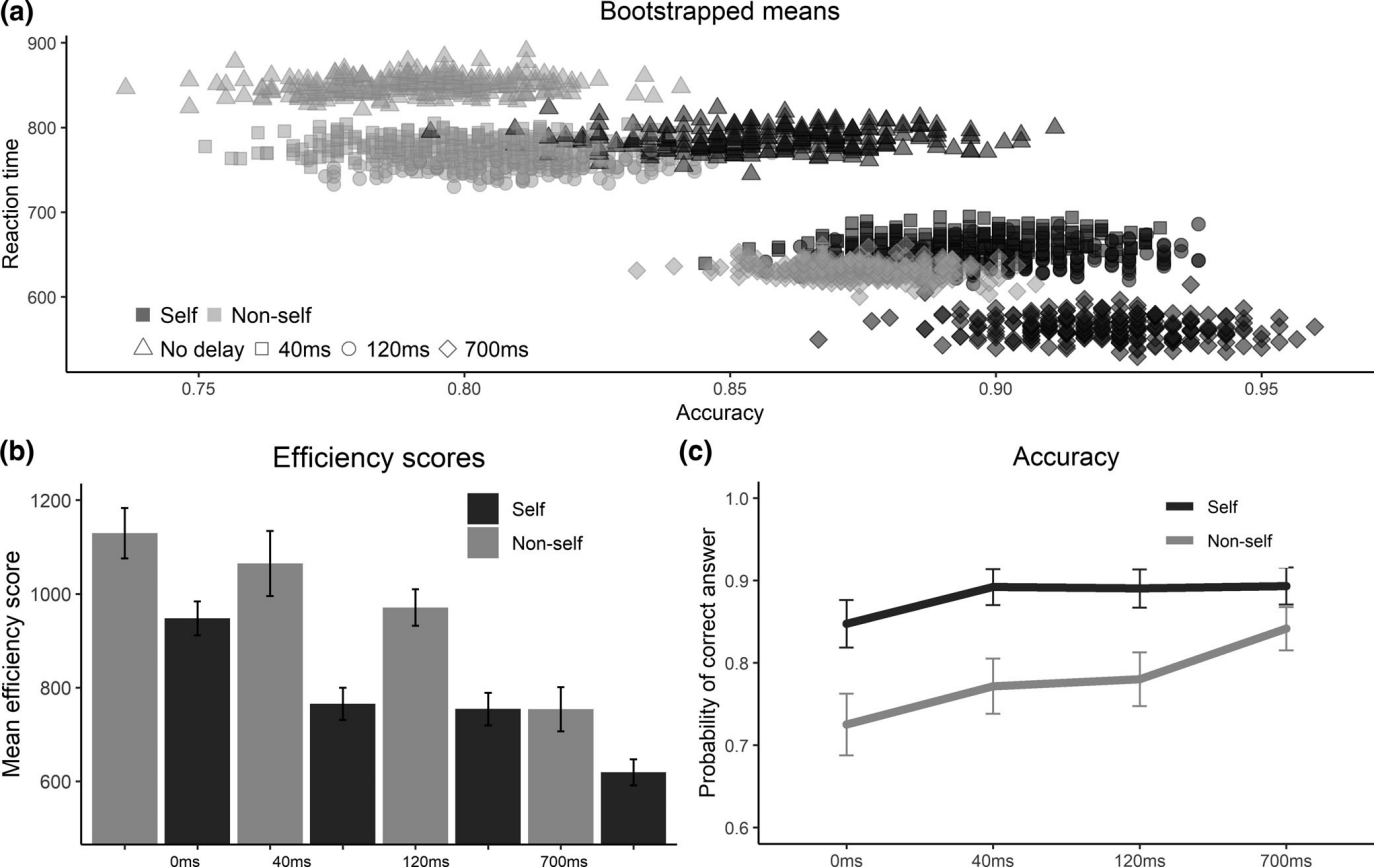
Effects of self and temporal delays. (a) Bootstrapped mean values of accuracy and reaction time, using 250 samples per condition sampled with replacement. (b) Mean efficiency scores (reaction time divided by accuracy), bars indicate standard errors. (c) Estimates from the logistic mixed model regression of the probability that a response is correct, separately for self and nonself over the different delays. Bars represent standard errors of the predictions

To elucidate the way in which the SPE changes over the delays in self and nonself, we first calculated bootstrapped means similarly as before. These are presented in Figure 3a. We calculated mean efficiency scores for each participant for each delay condition separately for self and nonself. The means are plotted in Figure 3b. We tested the difference in efficiency scores between self and nonself conditions (SPE) for each of the delays using *t*‐tests with Bonferroni correction. The SPE was significant at all but the longest delay condition (no delay: Δ = 198.6, *t* = 3.5463, *p* = .0052. 40 ms: Δ = 296.5, *t* = 4.09, *p* = .0012. 120 ms: Δ = 213.9, *t* = 6.02, *p* < .0001. 700 ms: Δ = 216.5, *t* = 2.43, *p* = .0822). These values do not indicate a clear trend of the SPE over the delays.

To test whether the SPE on accuracy and RT separately changed with the delays, we looked at the interaction effect of the Self and Delay variables in the LogMM and LMM, respectively. The average marginal SPE effects per delay are presented in Figure 3a. While a Wald test does not indicate a significant interaction of the SPE and the delays for accuracy (*χ* ^2^[3] = 3.01, *p* = 0.390), Figure 3c indicates a decreasing trend for the SPE across delays. However, the differences in SPE in the delays compared to no delay are not significant due to the large variance relative to the estimates, Table 1 provides the estimated differences in marginal effects of the self and their standard error. A Wald test shows that the interaction of the delays with the SPE for RT is just significant (*χ* ^2^[3] = 8.09, *p* = .0443). See Table 1 for the pairwise contrasts.

**TABLE 1:** Pairwise contrasts of self‐prioritization effect (SPE) between delays

Delay contrast	ΔSPE on accuracy	*SE*	*p*	ΔSPE on reaction times	*SE*	*p*
0 ms–40 ms	0.150	0.253	n.s.	−39.22	21.8	.0724
0 ms–120 ms	0.084	0.263	n.s.	−38.45	22.3	.0840
0 ms–700 ms	−0.290	0.265	n.s.	9.155	22.2	n.s.

*Note:* Pairwise contrasts of SPE under different delays based on mixed model regressions on accuracy and reaction time respectively.

### 3.4 Psychological level of self IV: Interindividual variability in interaction of self and delays

**FIGURE 4:**
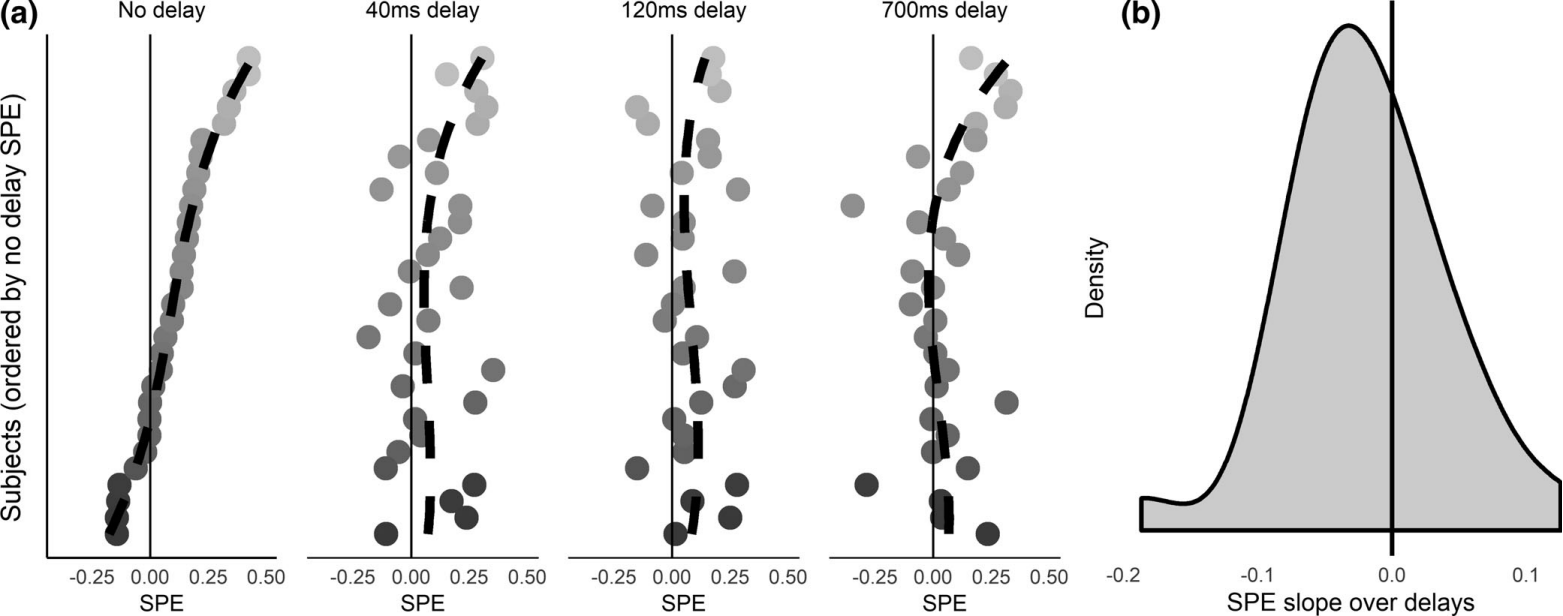
Individual variation in changes of self‐prioritization effect on accuracy over the delays. (a) Plots of each subject's self‐prioritization effect on accuracy separately plotted for each delay condition. The *x*‐axis can be read as the added probability of a correct answer due to the self. The *y*‐axis indicates each individual subject, all plots are sorted on the self‐prioritization effect in the no delay condition to illustrate how participant's self‐prioritization effect changes from the no delay condition to conditions with delays. Dashed black lines are trend lines which illustrate that the pattern from the no delay condition is not carried over to the delay conditions. (b) Density plot of the power‐law exponent (PLE) slopes. PLE slopes refer to the slopes of linear fits to each participant's SPE values. The slopes of these lines are used for the correlational analysis with EEG data

We observed considerable intersubject variance in the self‐delay interaction on accuracy, as evidenced by the standard errors in Table 1, which was thus subjected to further analyses on the single subject level (Figure 4). The *y*‐axis of all graphs in Figure 4a are ordered on the SPE, that is, the difference in accuracy between the self and nonself conditions, of individual subjects in the no delay condition: the figure shows high interindividual variation in the no delay SPE (left) and how that variability is carried over to the SPE in delay conditions (from left to right). If the SPE was carried over from the no delay conditions to the delay conditions, for example, if participants with low SPE in the no delay conditions would also have a relatively low SPE in the delay conditions, then we would observe a diagonal line in all graphs in Figure 4a as we do in the graph of the no delay conditions. This is not the case as the graphs show that there is no group‐level pattern in how the SPE gets carried over to the different delay conditions. Hence, intersubject variability of SPE holds over all temporal delays for which reason it may be to a large extent an intrinsic feature rather than being extrinsic (in which case changes in SPE would be similarly modulated by changing extrinsic task requirements like the different delays for each participant).

To better quantify interindividual variation across the different conditions, we calculated for each individual subject the slope of linear fits through each participant's SPEs on accuracy in the matching conditions over time. This slope is an index of how each individual's SPE changes due to the temporal delays. If this slope is positive for a participant, it indicates that this participant's SPE increases with the longer delays. These slopes have a mean of −0.0196 and a SD of 0.0595. This indicates that on average the SPE on accuracy decreased over the delays, but that there is a significant amount of interindividual variation: 17 subjects had a negative slope while 14 subjects exhibited a positive slope (see Figure 4b). We similarly calculated slopes for the SPE on RT. These slopes were subsequently used as basis for the correlation of the psychological SPE‐delay effects with the neuronal PLE of resting‐state EEG.

### 3.5 Cognitive function I: Relation of working memory to self and delays

To test the effect of working memory on efficiency, we first calculated overall efficiency scores for each participant separately and then performed a median split based on the working memory scores. We excluded one participant for who the working memory test failed due to a technical error. A two‐sample *t*‐test indicates that the participants in the high working memory group responded more efficiently overall (Δ = 114.5, *t* = 2.610, *p* = .0144). Next, we calculated the average SPE on efficiency for each participant separately and then compared these using *t*‐tests. The SPE on efficiency scores was not different for the participants in the high and low working memory groups (Δ = −11.24, *t* = −0.503, *p* = .6186).

We included the main effect of working memory and the interactions with the SPE and the delays as covariates in the LogMM. The results show a significant positive main effect of working memory on accuracy (*β* = .387, *SE* = 0.171, *p* = .024). Participants with higher working memory scores responded more accurately overall. However, the interaction with the SPE was not significant (*β* = .006, *SE =* 0.091, *p* = .946), nor was the interaction with the delays.

Different from accuracy, we find that working memory does not affect participant's overall RT (*β* = −2.05, *SE* = 22.8, *p* = .929). Nor do we find a significant interaction of working memory with the SPE (*β* = −2.39, *SE* = 7.89, *p* = .762).

Together these results show the effect of the self is not related to an individual's working memory capacity. It does seem that working memory affects accuracy overall, but not RTs; participants higher in working memory capacity respond more accurately regardless of the specific stimuli. Importantly, the data show that it holds for both self and nonself conditions; the SPE is thus not related to working memory effects.

### 3.6 Cognitive function II: Relation of learning effects to self and nonself

To see whether learning impacts behavior, we looked at how it changes during a block, since each block new associations were learned. We first calculated overall efficiency scores (i.e., independent of self and matching) for each participant separately for the first and last 50 trials in each block, a paired *t*‐test shows that participants were more efficient in the second half of the blocks (Δ = 44.51, *t* = 4.16, *p* < .0001). Next we calculated the SPE on efficiency for each participant separately for the first and latter half of the blocks. Paired t‐tests show that there is no significant difference for the SPE (Δ = −31.06, *t* = −1.82, *p* = .079), indicating that it did not change over the course of the experiment.

Next, to test the effect of learning on RTs and accuracy separately, we used a variable indicating the trial number within a block. We added both the main effect and the with the self to the LogMM and LMM regressions.

We find a significant main effect of trial number on accuracy (*β* = .189, *SE* = 0.082, *p* = .021), but no significant interaction effects. Participants become more accurate during a block, regardless of stimulus type.

The story for RTs is different: there is both a significant main effect of trial number (*β* = −31.2, *SE* = 8.09, *p* < .001) and a significant interaction effect with the SPE (*β* = 15.86, *SE* = 7.75, *p* = .041). To investigate this interaction, we calculated the average marginal effects (AME) of trial number separately for the self and nonself trials. The effect of trial number is highly significant for the nonself trials (*AME* = −20.8, *SE* = 4.71, *z* = −4.08, *p* < .001), but it is not for the self trials (*AME* = −4.90, *SE* = 6.14, *z* = −0.798, *p* = .43). So, learning affects RTs on nonself trials, but not on trials involving the self.

Taken together, our results show an overall or global learning effect that applied to all stimuli in an equal way: participants are quicker and more accurate at the end of a block. There was no learning effect specific to the self conditions, learning only has a specific impact on RTs of nonself trials.

### 3.7 Temporal integration on neuronal level I: PLE and ACW mediate delay effects on the SPE

**FIGURE 5:**
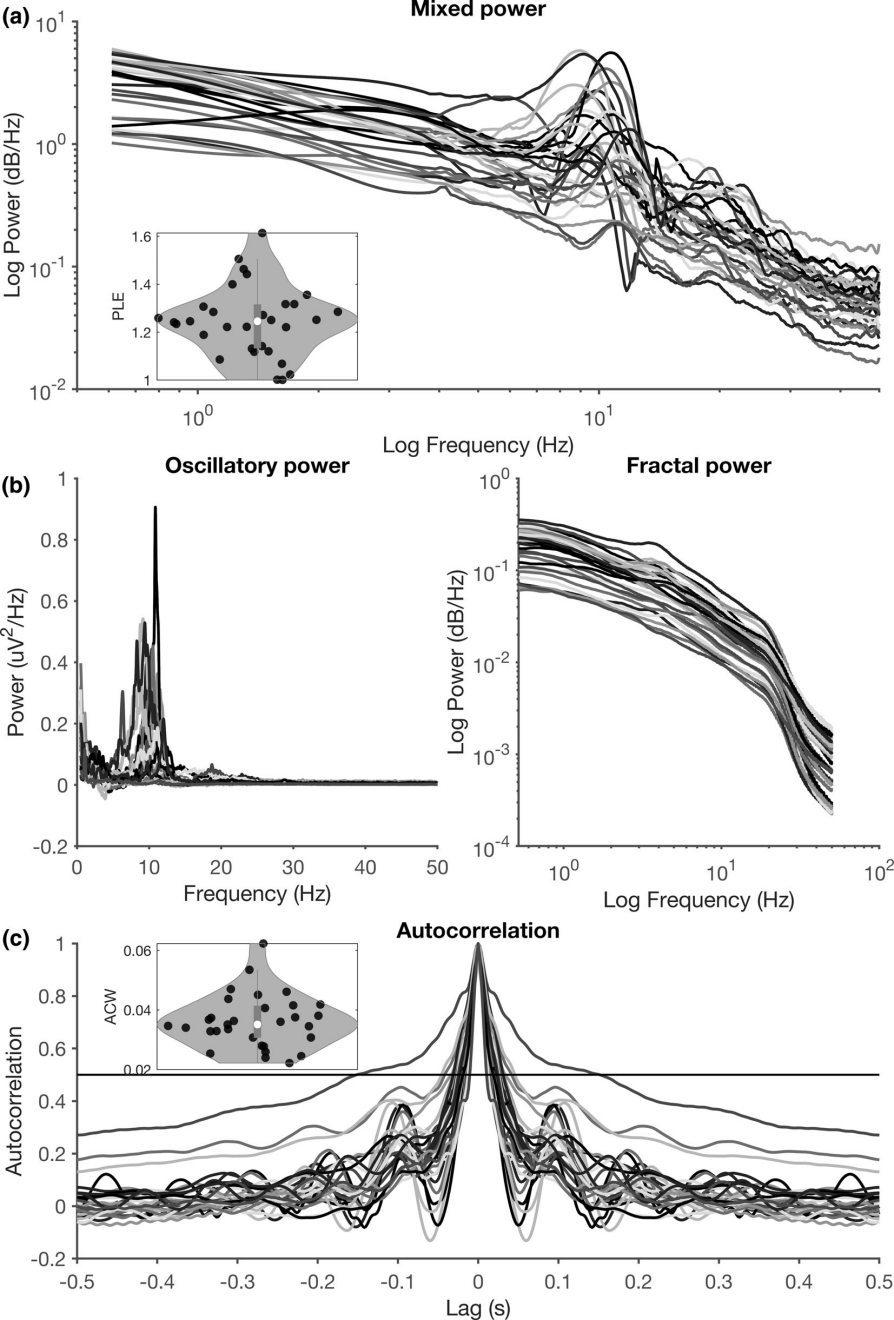
Across‐subject variability in power spectra and autocorrelograms. (a) Resting‐state power spectra for each individual subject. The distribution of power‐law exponents is plotted in the inset. (b) IRASA decomposition of the power spectrum into oscillatory (left) and fractal (right) components. Oscillatory components are plotted on a linear scale (as no a priori logarithmic structure is present), while fractal components are plotted on a log–log scale. (c) Resting‐state autocorrelograms for each individual subject (each subject is a color). The threshold of 0.5 is plotted as a horizontal line, and the distribution of resulting autocorrelation windows (see Section 2) is plotted in the inset

In a first step, we calculated resting‐state ACW and PLE for all electrodes based on the mixed‐spectrum EEG signal. This yielded typical ACW and PLE values in all subjects. As in previous EEG (Wolff et al., 2019) and fMRI (Huang et al., 2016) studies, we observed substantial interindividual variability in ACW (*M* = 0.0362, *SD* = 0.0089), mixed‐spectrum PLE (*M* = 1.24, *SD* = 0.145), and fractal‐spectrum PLE(*M* = 1.05, *SD* = 0.1175; see Figure 5a,b).

**FIGURE 6:**
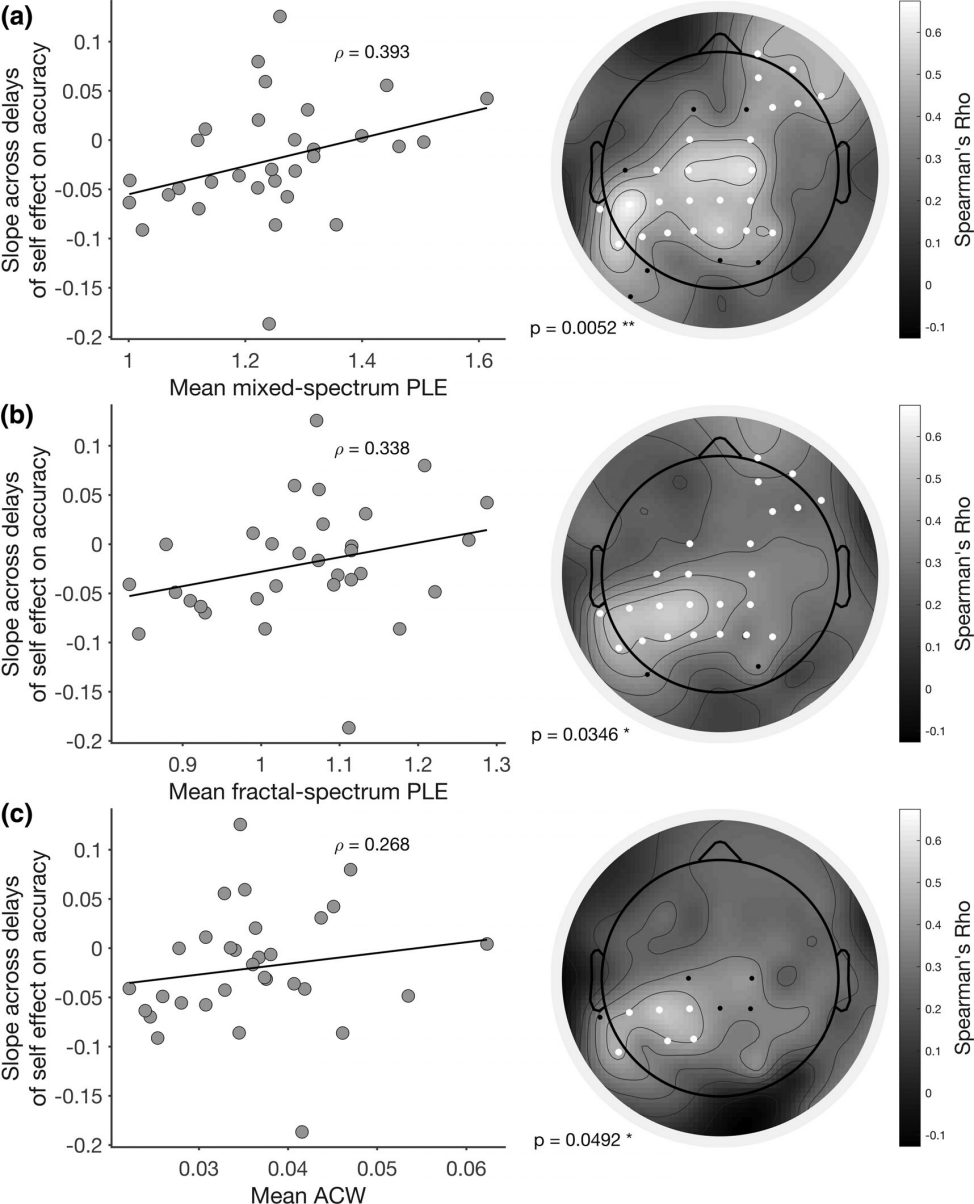
Correlation of EEG measures with slope across delays of self‐prioritization effects on accuracy (self‐prioritization effect [SPE] slope). (a) Correlation of mixed‐spectrum power‐law exponent (PLE) with SPE slope. (b) Correlation of fractal‐spectrum PLE with SPE slope. (c) Correlation of autocorrelation window with SPE slope. Scatter plots (left) show mean values across electrodes, and Spearman correlation coefficients using these mean values. Topographies (right) show the correlation coefficients across electrodes. White electrodes were included in a significant cluster (*p* values listed in figure), while black electrodes were significant at *p* < .05, but not following the cluster‐based correction

Next we correlated the mixed‐spectrum and fractal‐spectrum PLE, as well as the ACW, with the SPE slope over the different delays. That showed significant correlations of both resting‐state PLE and ACW with SPE slope for accuracy: the higher the PLE and the longer the ACW, the steeper the SPE slope across the different delays. Hence, higher PLE and longer ACW went along with stronger and thus more positive SPE effects over the longer delays (mixed‐spectrum PLE: *ρ* = 0.393, *p* = .0052; fractal‐spectrum PLE: *ρ* = 0.338, *p* = 0.0346; ACW: *ρ* = 0.268, *p* = .0492; see Figure 6). To rule out cognitive effects, we also included working memory scores as a co‐variate in these correlations: mixed‐spectrum PLE remained significant (*ρ* = 0.357, *p* = .0170), while fractal‐spectrum PLE and ACW remained marginal (*ρ* = 0.286, *p* = .0504 and *ρ* = 0.286, *p* = .0772, respectively). Finally, no correlation of SPE slope on RT with either PLE or ACW was significant.

Together, these data suggest that interindividual differences in temporal integration on the neuronal level of the brain's resting‐state activity, that is, PLE and ACW, are related to interindividual differences in changes of the SPE due to temporal delays on the psychological level, that is, the SPE slope. This temporal relationship of neuronal and psychological levels holds especially for the scale‐free, that is, fractal, component of the neuronal signal and cannot be traced psychologically to the cognitive components we measured, working memory, and learning.

### 3.8 Temporal integration on neuronal level II: Cortical midline structures

**FIGURE 7:**
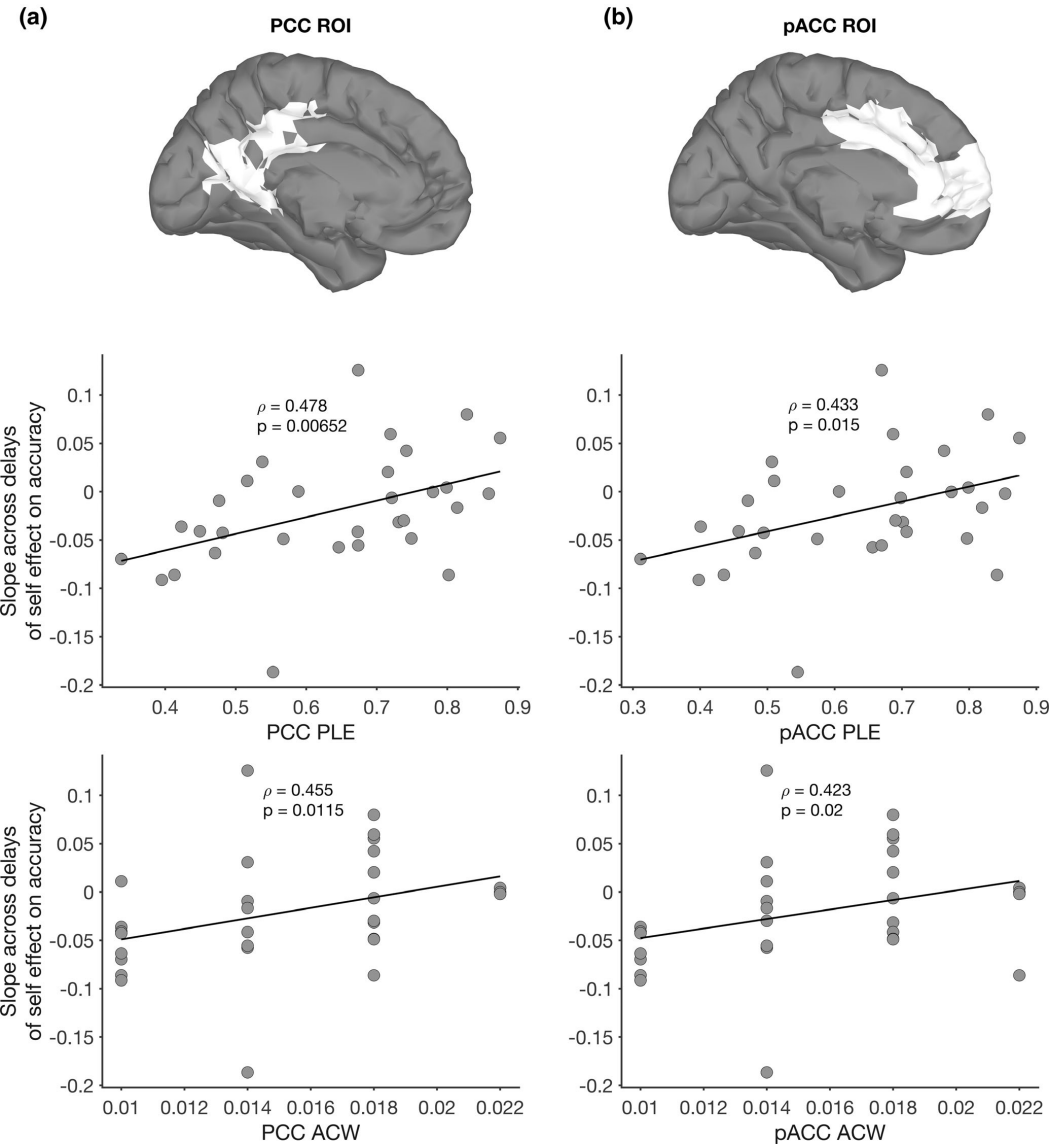
Regions of interest (ROI) definitions and correlations with self‐prioritization effect (SPE) slope for (a) PCC and (b) pACC PLE and autocorrelation window (ACW). Top panel shows the ROI definitions, according to Brodmann areas, interpolated onto a standard cortical surface in Brainstorm (Tadel, Baillet, Mosher, Pantazis, & Leahy, 2011). Bottom two panels show the correlation of mixed‐spectrum PLE and ACW values in each region with the slope across delays of the SPE on accuracy (SPE slope). Rho and *p*‐values correspond to Spearman's rank correlation

Finally, we conducted the same analyses in regions implicated in self‐related processing, pACC and PCC, as well as in control regions, the motor and visual cortex. This yielded significant correlations of SPE slope with mixed‐spectrum PLE and ACW in the pACC and PCC ROIs (see Figure 7). We found that one participant was an outlier (more than two standard deviations from the mean) in both pACC ACW and PCC ACW, her data are excluded from the results presented in Figure 7. The removal of this outlier did not affect our results (correlations including outlier: PCC ACW: *ρ* = 0.462, *p* = .00889; pACC ACW: *ρ* = 0.434, *p* = 0.0147). In contrast with the pACC and PCC, no significant correlations were present for the visual and motor cortices (Tables 2 and 3). However, a correlation was found in the somatosensory cortex with PLE as well; because of this, and the inherent low spatial resolution of source‐localized EEG, we refrain from making strong inferences on the specific regions involved in this effect. All correlations mentioned above survived partial correlation analysis when including working memory scores as a covariate (see Tables 2 and 3).

**TABLE 2:** Spearman and partial correlations of autocorrelation window with self‐prioritization effect slope in five regions of interest (ROIs)

	Spearman correlation		Partial correlation	
ROI	*R*	*p*	*R*	*p*
PCC	.462**	.00889	.470**	.00882
pACC	.434*	.0147	.453*	.0120
Visual cortex	.203	.273	.205	.277
Motor cortex	−.0200	.915	−.0238	.901
Somatosensory cortex	.231	.212	.236	.209

*Note:* ROIs estimated with eLORETA. Partial correlations are controlled for working memory score.

^*^ *p* < .05.

^**^ *p* < .01.

**TABLE 3:** Spearman and partial correlations of mixed‐spectrum power‐law exponent with self‐prioritization effect slope in five regions of interest (ROIs)

	Spearman correlation		Partial correlation	
ROI	*R*	*p*	*R*	*p*
PCC	.478 **	.00716	.481 **	.00716
pACC	.433 *	.0150	.437 *	.0157
Visual cortex	.329	.0705	.330	.0749
Motor cortex	.189	.308	.190	.314
Somatosensory cortex	.551 **	.00130	.552 **	.00157

*Note:* ROIs estimated with eLORETA. Partial correlations are controlled for working memory score.

^*^ *p* < .05.

^**^ *p* < .01.

Taken together, these results show that higher PLE and longer ACW, both of which index the scale‐free relationship of slow‐ and fast‐frequency components of the EEG signal, are related to a stronger SPE on accuracy with longer delays as compared to shorter delays or no delay. Participants with high PLE and long ACW values have a self‐bias that increases when delays become longer. This relationship is particularly strong for the pACC and PCC, areas that have been previously implicated in self‐related processing (Northoff et al., 2006; Northoff & Bermpohl, 2004; van den Meer et al., 2010; Murray et al., 2015). Working memory does not seem to affect this relationship, which marks it as primarily dynamic and temporal rather than being dependent on working memory or deliberate, cognitive processing.

## 4 DISCUSSION

We investigated temporal integration of self on the psychological level and how it is related to temporal integration on the neuronal level of the brain's resting‐state. First, we observed on the psychological level that the SPE was present across different temporal delays inserted by us in the perceptual matching task of Sui and colleagues (Sui et al., 2012, 2013; Sui, Yankouskaya, & Humphreys, 2015; Yankouskaya et al., 2020). Importantly, we analyzed accuracy separately from RTs, and found a different pattern of effects. Moreover, we showed that the SPEs of specifically the self‐specific stimuli over the different temporal delays could not be accounted for by cognitive effects related to working memory or learning.

Secondly, we demonstrated that interindividual variation in the SPEs of self‐specific stimuli over the different temporal delays was correlated with the interindividual variation in the PLE and ACW of resting‐state EEG. Importantly, this relationship was not affected by cognitive functions, and was most prominent in the midline regions pACC and PCC.

Taken together, our data show that temporal integration on the psychological level of self (as tested for in the delays of the self‐matching task) is related to temporal integration on the neuronal level of the brain's spontaneous activity (as indexed by PLE and ACW). We therefore conclude that temporal integration mediates the connection or link of neuronal and psychological levels of self, thus reflecting what recently has been described as “common currency” (Northoff et al., 2020).

### 4.1 Temporal integration on the psychological level of self

We confirmed and replicated the SPE in the matching conditions in the no delay condition as observed in the various studies by Sui and colleagues (Sui et al., 2012, 2013, 2015; Yankouskaya et al., 2020). We extend these findings by our observation that the newly inserted delay conditions in the self‐matching paradigm did not change the SPE on accuracy at the group level. Hence, we observed SPE effects in all delays, that is, participants responded on average significantly more accurately to the self trials compared with the nonself trials across all delays. This strongly suggests that the SPE from the no delay condition is carried over to the temporal domain such that it holds also during the delays. Such temporal extension of the SPE means that subjects can integrate label and shape across different time points, that is, over the delays. That, in turn, requires temporal continuity on the psychological level of self‐specificity, thus giving support to what recently has been described as self‐continuity (Ersner‐Hershfield et al., 2009; Huang et al., 2016; Northoff, 2017; Wolff et al., 2019).

While we found that the SPE on average is present even when delays are inserted, a group‐level analysis does not tell the full story. There is substantial interindividual variation in both the SPE itself and how it changes across the delays. Moreover, these are not related, the magnitude of the SPE of a subject in the no delay condition seems to be unrelated to whether the SPE increases or decreases due to delays. Hence the SPE seems to be largely an intrinsic or trait feature of a subjects rather than being determined by the extrinsic task demands, that is, the state. How an individual responds to self‐related stimuli across different timescales is personalized, in other words, it is dependent on that individual's intrinsic or trait features: our data suggest that this can be traced, in part, to resting‐state brain activity. The notion of an intrinsic temporal trait feature characterizing the self is in line with findings showing that differences in how people experience their self extending over time severely impacts their behavior (Ersner‐Hershfield et al., 2009), and with the clinical view that holds the self as being a central criterion by which to assess personality (Scalabrini et al., 2018).

Importantly, we did not observe any relation of interindividual variation in working memory to the interindividual variation in the impact of the temporal delays on SPE. This does not support the hypothesis that the temporal delay effects in SPE are mediated primarily by a cognitive function like working memory (see Janczyk, Humphreys, & Sui, 2018). However, our data do not rule out working memory effects as we did not specifically test for all the different forms of working memory, nor for memory effects related to our specific stimuli. We did test for learning effects and found accuracy to increase within blocks of trials. However, these learning effects were observed in both self‐ and nonself‐specific conditions without any specific relation to the temporal SPE effects. This makes learning a rather unlikely source of the SPE.

### 4.2 Temporal integration links neuronal and psychological levels

The psychological effects suggest a primarily dynamic (i.e., temporal rather than cognitive) basis of the temporal SPE effects. We therefore analyzed the relationship between the psychological SPE and temporal integration on the neuronal level as indexed by PLE and ACW. Correlation analyses show close relationship of psychological and neuronal indices of temporal integration: the higher the PLE and the longer the ACW in EEG resting‐state, the stronger the SPE increased over increasing delays.

The relationship of scale‐free properties of neuronal activity with the temporal delay effects on the SPE suggests that temporal integration on the neuronal level shapes temporal integration on the psychological level of self. We therefore propose that the relationship between neuronal ACW/PLE and psychological SPE delay effects may be mediated in a temporal way—temporal integration could provide the “common currency” (Northoff et al., 2020) of neuronal and psychological levels. That is further supported by our observation that cognitive functions like working memory and learning neither modulated SPE delay effects on the psychological level nor co‐varied with the ACW/PLE–SPE delay relationships.

Finally, given our eLORETA results, temporal integration mediating neuronal and psychological levels seems to be particularly strong in cortical midline structures like pACC and PCC. In contrast, we did not observe such a strong relationship in primary regions like visual and motor cortices. We did observe a significant relationship in the somatosensory cortex, but only for the PLE. As such, this correlation may not reflect the influence of scale‐free activity as much as the unique noise sources and biases that affect the PLE estimation; it is for exactly this reason we included both PLE and ACW as measures. Our findings are in line with previous studies using fMRI (Huang et al., 2016) and EEG (Wolff et al., 2019), with both showing a relationship of resting‐state PLE in pACC and PCC with self‐consciousness. The findings presented here are also in accordance with studies that show resting‐state PLE in midline regions to modulate task‐evoked activity in response to self‐related stimuli (Scalabrini et al., 2017, 2019). As such our findings agree with the well‐known association of these regions with the self (Murray et al., 2015; Northoff & Bermpohl, 2004; Northoff et al., 2006; van den Meer et al., 2010). Accordingly, our data lend further support to the observed “rest‐self overlap” (Bai et al., 2016) and “rest‐self containment” (Northoff, 2016) in specifically the cortical midline structures (Qin & Northoff, 2011). This “overlap” or “containment” suggests a “basis model of self‐specificity” as distinguished from a higher‐order cognitive model of self (Northoff, 2016). That we found working memory and learning not to affect our results supports this basis model of the self. Moreover, our findings extend this model to the temporal domain by showing that temporal integration in the midline regions is related to temporal integration on the psychological level of self. More generally, we provide further evidence for the notion that individual differences in resting‐state brain activity, intrinsically related to the self, are crucial in understanding how individual differences arise in behavior (Scalabrini et al., 2018).

### 4.3 Temporal pooling as mechanism of temporal integration

The exact mechanism that connects temporal integration from neuronal to psychological levels remains unclear. Temporal integration is closely related to what recently has been described as “temporal pooling” that refers to how different inputs over a specific time window are summed and integrated within one and the same activity (of a neuron or region; Himberger et al., 2018, p. 163). Such temporal pooling and integration make the neural response robust to changes in the inputs' timing and allows for combining past and present inputs into one and the same pattern of activity. This integration is required for the self to extend over longer timescales.

Temporal pooling also allows for the summation or integration of different inputs from different sources—from internal cognitive inputs, interoceptive bodily inputs, and exteroceptive inputs from the world—at different points in time. Hence, such temporal pooling would provide a mechanism for cognition, the body, and aspects of the world to be integrated into the self.

Our self‐matching paradigm with the newly inserted temporal delays requires such temporal pooling: subjects had to integrate external inputs presented at different time points. The better they can pool and sum the two temporally delayed inputs, that is, shape and label, the higher their SPE effects over the temporal delays.

We measured temporal integration in the frequency (PLE) and time (ACW) domains. We can apply these mathematically equivalent viewpoints to temporal pooling as well. The more different time points in neuronal activity are correlated over longer stretches of time (time‐domain lens; ACW), the more likely it is that inputs arriving at different points in time impact neuronal activity in such a way that they are pooled and integrated in the same pattern of neuronal activity (see Himberger et al., 2018). Looking through the frequency‐domain lens, slower frequencies have long cycle durations, which makes brain activity with strong power in these frequencies (PLE) ideally suited for pooling and integrating different stimuli at different time points together (He & Raichle, 2009; Himberger et al., 2018; Honey et al., 2012).

We thus propose, albeit tentatively, that temporal pooling of inputs is a candidate mechanism of temporal integration on the psychological level of self. That is, temporal pooling is in part responsible for the manifestation of a self that is extended over time and over different sources (cognition, body, and world) of neuronal activity. Moreover, it leads to self‐coherence as activity related to different aspects to the self are pooled into the same pattern of activity. Such a mechanism would agree with the processing advantage implied by the SPE (Sui & Humphreys, 2015) and in particular with the proposal of Scalabrini and colleagues that the current temporal structure of spontaneous brain activity predisposes or aligns an individual's response to external self‐related stimuli (Scalabrini et al., 2017, 2019). The neuronal activity of these self‐related stimuli may be pooled into a pattern of brain activity that extends over longer timescales, whereas nonself‐related stimuli would not, or to a lesser extent, benefit from this particular processing mechanism.

Taken together, we suggest that temporal pooling may underlie the observed temporal integration on both neuronal and psychological levels. We hypothesize that temporal pooling is a mechanism that mediates the temporal integration on both the neuronal and psychological level. Temporal pooling and thus temporal integration can therefore be considered an example of what was recently described as “common currency” of neuronal and psychological levels (Northoff et al., 2020). However, more research is needed to lend further support to this hypothesis. In particular, future research should directly test for the temporal integration of inputs from multiple sources, that is, from cognitive processes, from the own body, and from the external world.

### 4.4 Limitations

Some limitations of this study need to be considered. First, using EEG without adjusting for individual anatomy can yield localization errors, and as such our localization results can at best be considered an approximation. Second, due to practical constraints, we could not include a more fine‐grained continuum of different time delays in our self paradigm. That precludes a more comprehensive parametric mapping of self‐delay interaction effects. Third, we here focused on linking resting‐state measures of scale‐free activity to psychological effects of self‐delay interaction; that excludes any relevant effects task‐evoked activity might have on the self‐delay interaction. Lastly, we only included a rather limited cognitive battery which, in future studies, may need to be complemented by more a comprehensive battery that includes tasks testing for individual differences in memory and executive functioning.

## 5 CONCLUSION

In conclusion, we here provide evidence for a relationship between temporal integration on both neuronal and psychological levels. Specifically, neuronal indices of temporal integration, for example, PLE and ACW, relate to psychological measures of temporal integration with respect to self, that is, SPE delay effects. We therefore tentatively propose that temporal integration on the neuronal level serves as template or blueprint (in yet unclear ways though) for temporal integration on the psychological level—temporal integration may serve as “common currency” of brain and self (Northoff et al., 2020). While we suggest a specific temporal mechanism like temporal pooling to mediate neuronal and psychological levels of temporal integration, future studies are warranted to substantiate this hypothesis and to investigate how the here suggested temporal mechanisms are related to cognitive ones such as memory or attention.

## DATA AVAILABILITY STATEMENT

The data that support the findings of this study are available from the corresponding author upon reasonable request.

## Supporting information


**Appendix** **S1.** Supporting Information
