# Supplementary material for: Temporal integration as “common currency” of brain and self‐scale‐free activity in resting‐state EEG correlates with temporal delay effects on self‐relatedness
Source: Hum Brain Mapp. 2020 Jul 22;41(15):4355–74. doi: 10.1002/hbm.25129 (PMC7502844; doi:10.1002/hbm.25129)
Supplement: Supplementary file 1 — Appendix S1. Supporting Information [file HBM-41-4355-s001.docx]

**Supplementary materials for Kolvoort et al. (2020)**

Results in different frequency bands

It has been proposed that fractal power spectra reflect multiple scale-free processes, which different scaling regimes in different frequency bands (Muthukumaraswamy and Liley, 2019; Nagy et al., 2017). With this in mind, we repeated our main analyses concerning the relationship between neuronal and psychological levels, i.e. correlations between ACW/PLE and SPE slope, in three different frequency bands: 0.5 – 5 Hz, 5 – 20 Hz, and 20 – 50 Hz. These results are presented below.

*
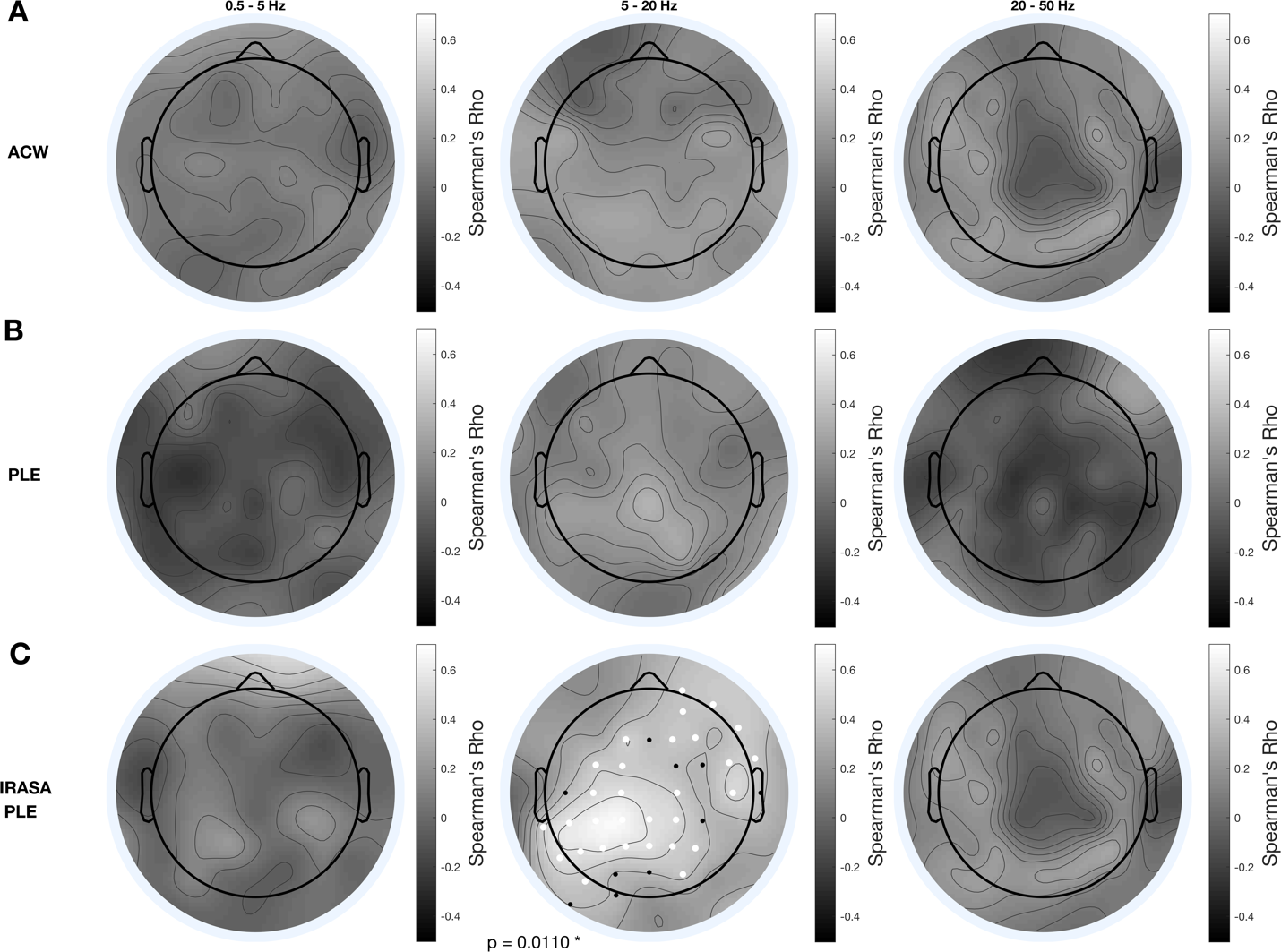
*

Figure S1. Results of correlations of (A) ACW, (B) mixed-spectrum PLE, and (C) IRASA/fractal-spectrum PLE with SPE slope (as in Figure 6). Topoplots show the scalp distribution of Spearman’s rho for the correlation with SPE slope. Electrodes in a significant cluster are labelled with white dots, while electrodes which were significant at the sensor level, but not following the permutation test, are indicated in black.

We find significant correlations only in the mid-frequency range for the IRASA power spectrum. This suggests that this mid-frequency range is most significant one for temporal integration: it may be that ACW and mixed-spectrum PLE don’t correlate due to the outsize influence of the alpha band in this range. Alternatively, given that the 20-50 Hz band is affected by filtering artefacts (see Figures S4 and S5), it may be that the 5-20 Hz range extends beyond 20 Hz, and reflects a general high-frequency power-law relationship. In either case, it appears that the relative absence of high-frequency power is particularly important for temporal integration.

Correlations between ACW, mixed-spectrum PLE, and IRASA/fractal-spectrum PLE

As mentioned in the main text, ACW and PLE are similar measures of fractal structure, and are equivalent in some cases. However, they may be differentially affected by noise, oscillations, and the various preprocessing steps applied to EEG signals. The figure below shows the intercorrelations between the three measures applied in the main text (ACW, PLE, and IRASA/fractal-spectrum PLE).


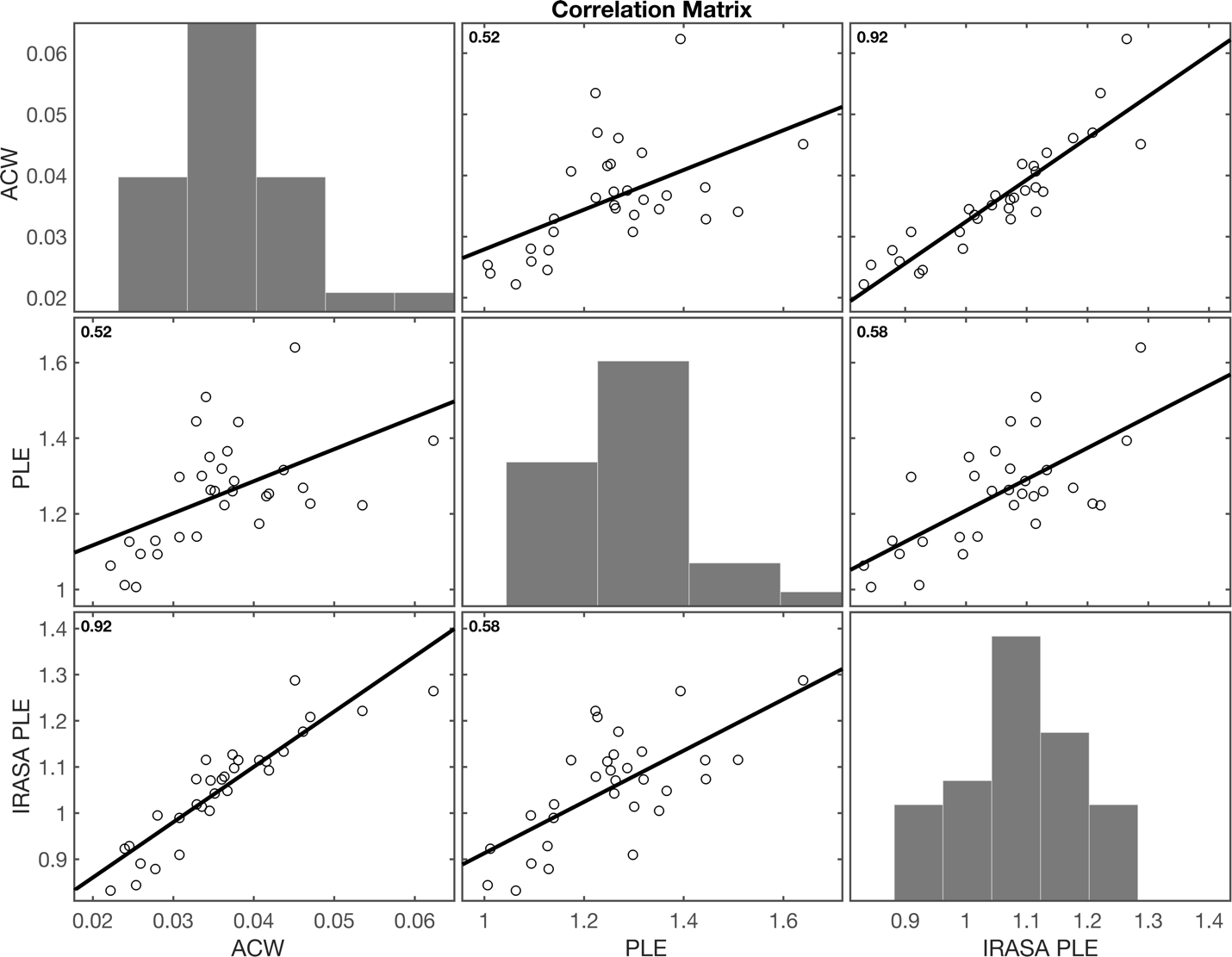


Figure S2. Spearman correlations between the three EEG measures used in the text. Correlations were assessed on the mean value of the measure over all electrodes. All correlations were significant with p < 0.01.

These results show that, as expected, the three measures of fractal structure are correlated with each other. However, these correlations are far from perfect, and as such it stands to reason that they are differentially affected by the various non-fractal processes in the EEG signal.

Partial correlations controlling for alpha oscillations

As mentioned above, the PLE and ACW estimators may be affected by non-scale-free processes. The most prominent of these is the alpha oscillation, occurring at roughly 10 Hz (Buszáki, 2006). While we have controlled for this by using the IRASA/fractal-spectrum PLE, we show here additional confirmation that the correlations of mixed-spectrum PLE and ACW with SPE slope are not affected by alpha. We present partial correlations of mixed-spectrum PLE and ACW with SPE slope, controlling for three alpha-related parameters: individual alpha frequency, individual alpha peak width, and individual alpha power. The first two were assessed using the methods of Corcoran et al. (2018), while the third was assessed using MATLAB’s *bandpower* function in the individually determined alpha band.


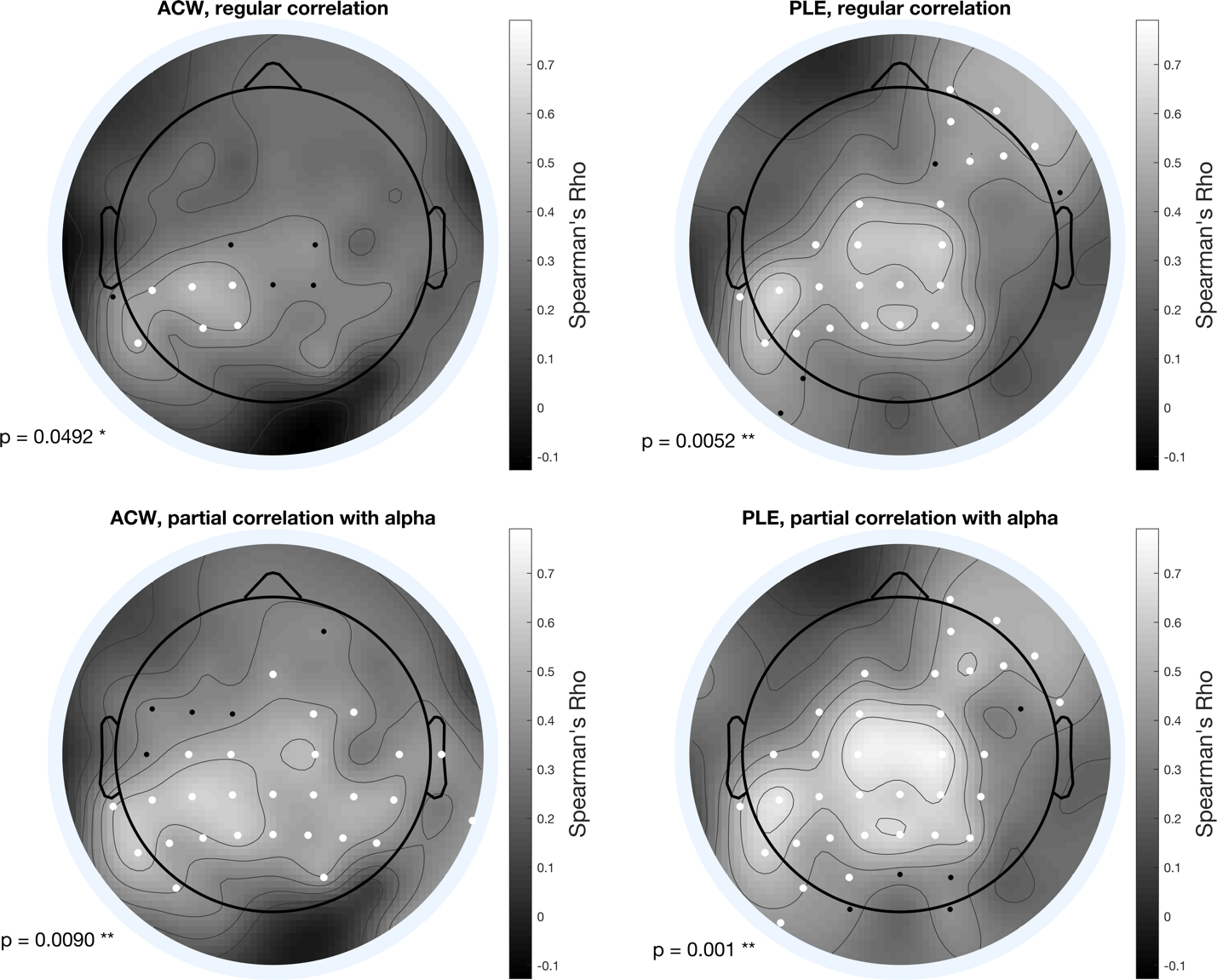


Figure S3. Results of partial correlations of ACW and mixed-spectrum PLE with SPE slope (as in Figure 6), controlling for individual alpha peak frequency, individual alpha peak width, and individual alpha power. Topoplots show the scalp distribution of Spearman’s rho for the partial correlation with SPE slope. Electrodes in a significant cluster are labelled with white dots, while electrodes which were significant at the sensor level, but not following the permutation test, are indicated in black.

The alpha band is the most prominent non-fractal process in the EEG power spectrum, and its presence may bias the estimation of mixed-spectrum PLE and ACW (IRASA/fractal-spectrum PLE should be unaffected, as the IRASA procedure separates the fractal power spectrum from the oscillatory). These results show that this alpha-related bias does not drive our main results: in fact, controlling for alpha-related parameters in the estimation improves the correlations of mixed-spectrum PLE and ACW with the SPE slope markedly.

IRASA filtering artifacts

In Figure 5 in the main text, one can see a sharp dip in the fractal PSD around 25 Hz. We show here with simulations that this reflects an artifact of the bandpass filtering applied to preprocess the data. We simulated 50 monofractal signals using the fBm/fGn model advocated in Eke et al. (2002). The fBm signals were generated using MATLAB’s *wfbm* function, and the fGn signals were generated by differentiating fBm signals. 21 seconds of each signal was generated, with a random β between 0 and 2. A lowpass (Figure S4) or highpass (Figure S5) FIR filter at various frequencies was applied in EEGLAB. The fractal PSD was estimated with the windowed IRASA method described in the methods section of the main text.


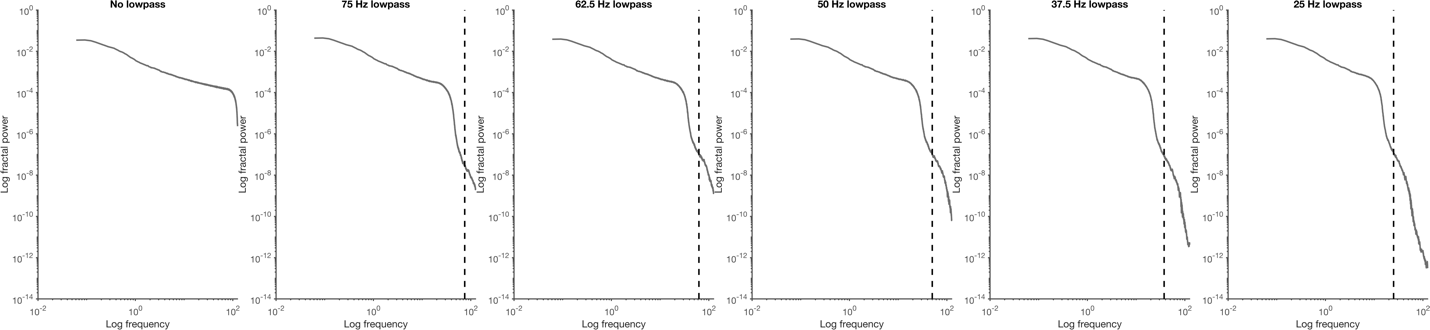


Figure S4. Spurious drops in the fractal PSD induced by low-pass FIR filters. Dashed line indicates the filter cutoff frequency.


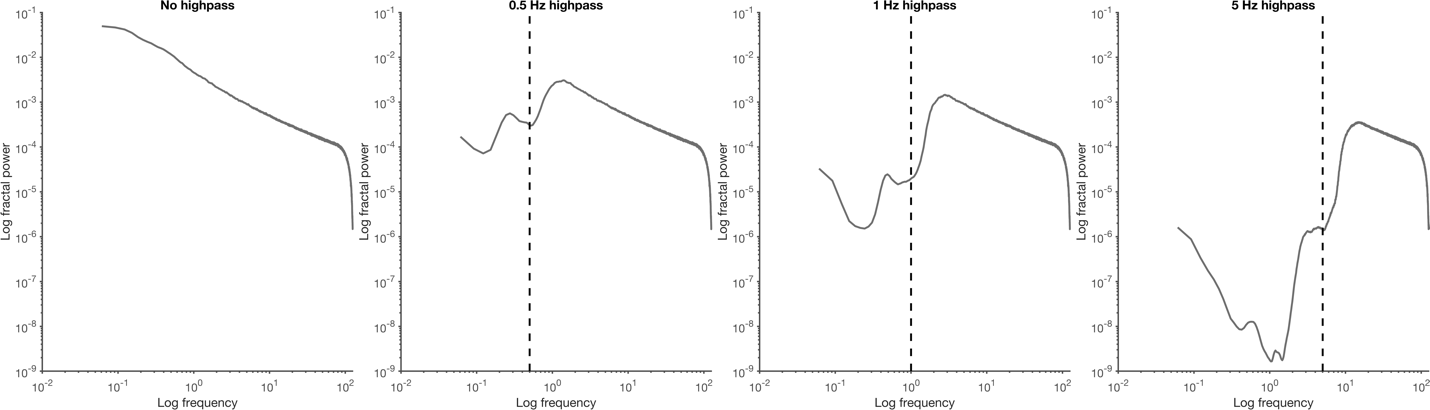


Figure S5. Spurious drops in the fractal PSD induced by high-pass FIR filters. Dashed line indicates the filter cutoff frequency.

These results clearly show that the application of an FIR filter introduces a spurious drop in the fractal PSD near the filter cutoff frequency. We have not yet investigated the reasons for this effect. However, the fractal power spectrum is calculated by taking the median PSD of many resampled signals, we suggest that small attenuations of the PSD related to the filter passband may result in outsized effects on the fractal component, as these drops make the signal no longer scale-free in these frequency ranges. This is the reason why the IRASA/mixed-spectrum PLE in the main text was fit over a restricted frequency range of 2-25 Hz.

**Additional references**

Corcoran, A. W., Alday, P. M., Schlesewsky, M., & Bornkessel‐Schlesewsky, I. (2018). Toward a reliable, automated method of individual alpha frequency (IAF) quantification. *Psychophysiology*, *55*(7), e13064.

Nagy, Z., Mukli, P., Herman, P., & Eke, A. (2017). Decomposing Multifractal Crossovers. *Frontiers in Physiology*, *8*.
